# Synthesis, Cytotoxicity and Antiproliferative Effect of New Pyrrole Hydrazones

**DOI:** 10.3390/molecules29235499

**Published:** 2024-11-21

**Authors:** Stanislava Vladimirova, Rossitsa Hristova, Ivan Iliev

**Affiliations:** 1 Department of Organic Synthesis, University of Chemical Technology and Metallurgy, 8 Kliment Ohridski Blvd., 1756 Sofia, Bulgaria; 2 Roumen Tsanev Institute of Molecular Biology, Bulgarian Academy of Sciences, 1113 Sofia, Bulgaria; rosi_hristova@hotmail.com; 3 Institute of Experimental Morphology, Pathology and Anthropology with Museum, Bulgarian Academy of Sciences, 1113 Sofia, Bulgaria; taparsky@abv.bg

**Keywords:** synthesis, pyrrole, hydrazones, antiproliferative effect, cytotoxicity

## Abstract

Novel pyrrole-based carbohydrazide (**1**) and hydrazones (**1A**–**D**) were synthesized, characterized, and subjected to spectroscopic studies. The hydrazones were obtained by reacting a pyrrole hydrazide with substituted pyrrole aldehydes. The initial carbohydrazide was prepared by selective hydrazinolysis of the obtained *N*-pyrrolylcarboxylic acid ethyl ester. The biological activity of the newly synthesized compounds was investigated in vitro on a panel of tumor and non-tumor cell lines. Mouse embryonic fibroblasts BALB 3T3 clone A31 were used in the safety test (BALB 3T3 NRU-assay). Antiproliferative activity was determined on keratinocytes (HaCaT) and melanoma (SH-4) cells by MTT dye reduction assay. The safety test of the compounds showed low cytotoxicity and absence of phototoxic potential. Among our novel pyrrole hydrazones, **1C** was the most selective (SI = 3.83) in human melanoma cells and exhibited very good antiproliferative activity (IC_50_ = 44.63 ± 3.51 μM). The cytotoxic effect of **1C** correlates with its ability to induce apoptosis and to cause cell cycle arrest in the S phase. In addition, the results show that hydrazones obtained by condensation with β-aldehydes are more bioactive than those obtained by condensation with α-aldehydes.

## 1. Introduction

Cancer represents a significant global public health challenge and stands as a prominent contributor to mortality worldwide [1,2,3]. There are more than 100 different types of cancers, each with its own characteristics, risk factors, and treatment options. Cancer is caused by the accumulation of genetic mutations over time, and these mutations can result from various factors, including exposure to carcinogens, genetic predisposition, lifestyle choices, infections, and other environmental factors [4,5]. Over time, the cure rate of patients has increased due to improved early diagnosis and more personalized treatments. Among these treatments are radiation therapy, surgery, immunotherapy, endocrine therapy, gene therapy, and chemotherapy, the latter being the most widely used either as monotherapy or in combination with other treatments [6]. However, resistance to chemotherapy in aggressive cancers has increased over time, which, together with the adverse effects chemotherapy causes, has led to the need for the development of new anticancer agents [7,8,9,10].

**Figure 1:**
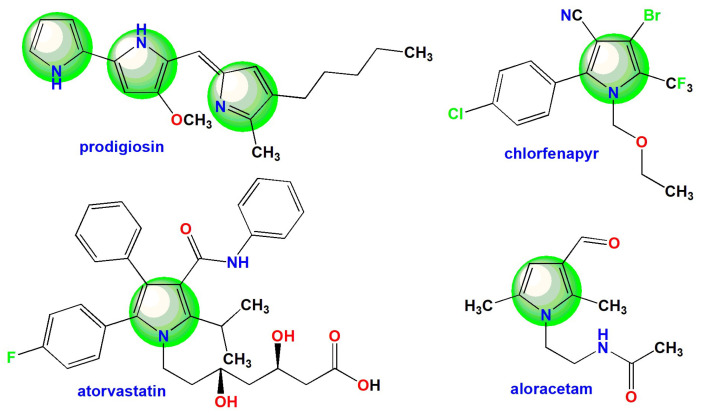
Marketed drugs containing pyrrole moiety.

**Figure 2:**
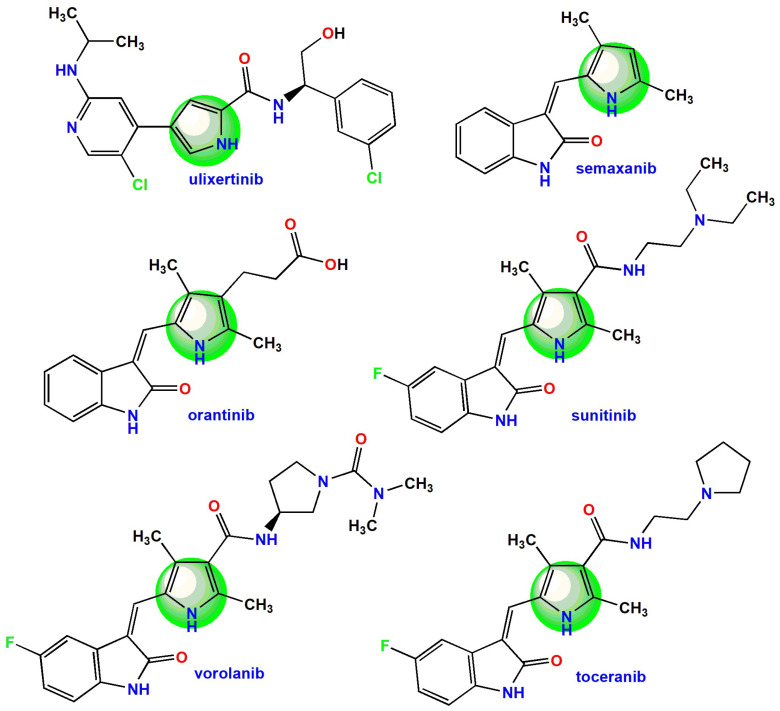
Marketed anticancer drugs containing pyrrole moiety.

Natural products have inspired the synthesis of a number of compounds with pharmaceutical applications, most of which are based on *N*-heterocyclic moieties—approximately 84% of the total number of molecules contain at least one nitrogen atom, and 59% contain at least one nitrogen heterocycle [11]. Among them, the pyrrole ring is one of the most studied heterocycles in the drug discovery process for several therapeutic areas [12,13], which is confirmed by the large number of pyrrole-based drugs that reach the market—prodigiosin (antibacterial), chlorfenapyr (insecticide), aloracetam (against Alzheimer’s), atorvastatin (antihyperlipidemic), etc. (Figure 1), including those with anticancer activity (Figure 2) [14]. In addition, this ring template is also found in several biomolecular structures such as chlorophyll [15], hemoglobin, myoglobin, cytochromes, vitamin B12, and bile pigments such as bilirubin and biliverdin [16,17].

**Figure 3:**
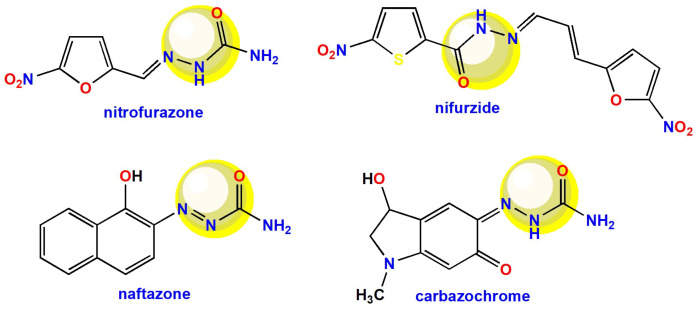
Marketed drugs containing hydrazone scaffold.

On the other hand, hydrazones are also an important class of organic compounds that possess diverse biological and pharmacological properties such as antimicrobial, anti-inflammatory, analgesic, antifungal, anti-tubercular, antiviral, anticancer, anticonvulsant, cardioprotective, etc. [18,19,20,21,22,23]. A number of derivatives containing the hydrazone pharmacophore (CO–NH–N=) moiety are used in therapy, such as nitrofurazone (antimicrobial), carbazochrome (antihemorrhagic), nifuroxazide (intestinal antibacterial), dantrolene (muscle relaxant), nitrofurantoin (antibacterial), nifuratel (antitrichomonal and antifungal), nifurzide (intestinal anti-infective), nifurtoinol (urinary anti-infective), naftazone (capillary stabilizing), azimilide (anti-arrhythmic), zorubicin (cytotoxic antibiotic) [24]. The structures of some representative pharmacologically active agents containing the hydrazone scaffold are shown in Figure 3.

Based on the significance of pyrrole and hydrazone scaffolds in drug design, providing relevant examples of marketed drugs, we focused our study on the synthesis of some pyrrole hydrazones in order to evaluate their antiproliferative activity against melanoma cells (SH-4) and keratinocytes (HaCaT). In addition, we also report the cytotoxicity and phototoxicity of the tested compounds against mouse embryonic fibroblasts (BALB 3T3 clone A31).

## 2. Results and Discussion

### 2.1. Chemistry

#### 2.1.1. Synthesis of *N*-Pyrrolylcarbohydrazide (**1**)

**Scheme 1:**
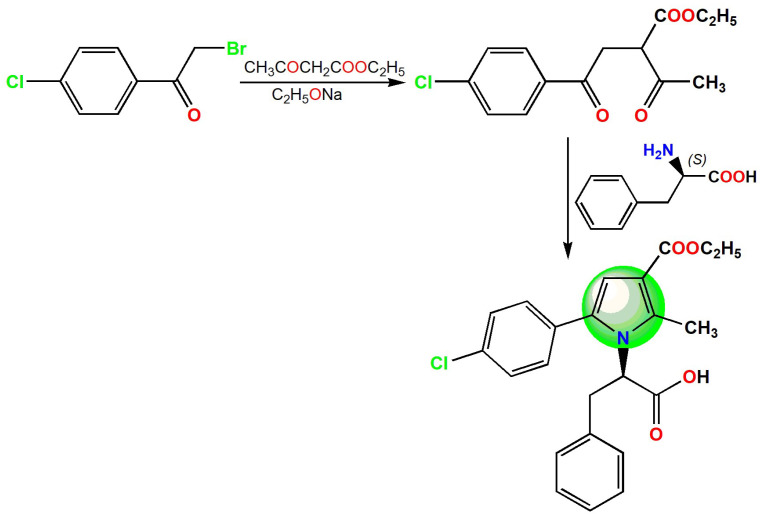
Synthetic access to the targeted *N*-pyrrolylcarboxylic acid.

The procedure for the synthesis of the targeted carbohydrazide started from the interaction of 2-bromo-4′-chloroacetophenone and ethyl acetoacetate in sodium ethylate media to obtain the 1,4-dicarbonyl compound. 2-bromo-4′-chloroacetophenone was used as a starting material to introduce a 4-chlorophenyl moiety into the target molecule. This structural moiety is present in the insecticide chlorfenapyr and in a number of previously synthesized-by-us compounds with a various biological activity [25,26,27]. The next step was the preparation of the target *N*-pyrrolylcarboxylic acid using the classical Paal–Knorr cyclization, as pointed out in Scheme 1. In this cyclization, L-phenylalanine was used as a primary amine. Many L-phenylalanine-derived compounds exhibit notable biological effects [28,29,30,31], so they have been widely used in the fields of food, medicine, spices, cosmetics, and agriculture [32]. In addition, a phenylalanine moiety is found in a number of molecules synthesized earlier [27,33,34,35,36].

The synthesis of the carboxylic acid and its characterization by spectroscopy and thin-layer chromatography was earlier described [37].

**Scheme 2:**
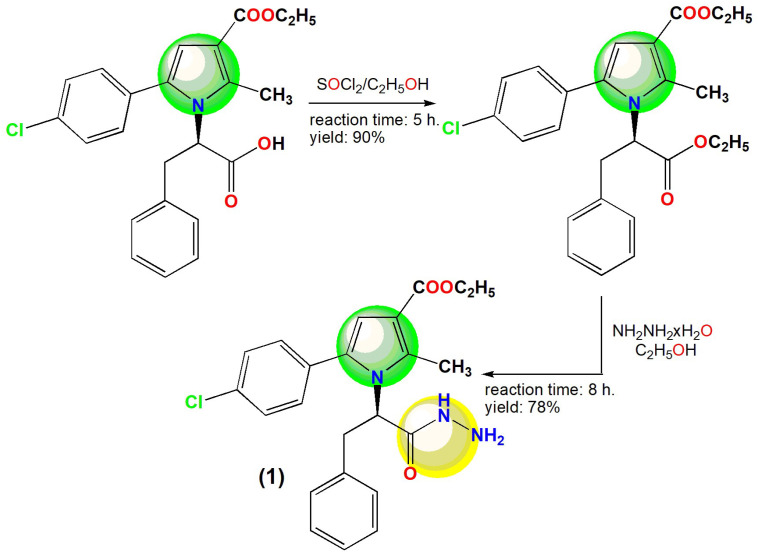
Synthetic access to the targeted *N*-pyrrolylcarbohydrazide (**1**).

The desired *N*-pyrrolylcarbohydrazide (**1**) was synthesized through selective hydrazinolysis of the intermediately prepared ethyl ester, as pointed out in Scheme 2. [38]. Its structure was identified through IR, ^1^H-NMR, ^13^C-NMR, and Mass spectra (see Supplementary Materials), and the purity was elucidated through HPLC and melting point.

#### 2.1.2. Synthesis of Pyrrole Hydrazones (**1A**–**D**)

**Scheme 3:**
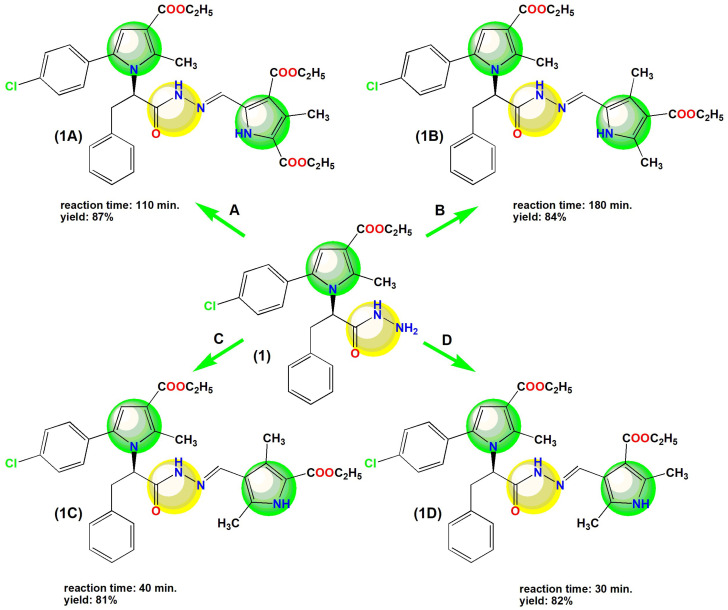
Synthesis of hydrazones (**1A**–**D**).

The targeted hydrazones were synthesized according to Scheme 3 by condensation between *N*-pyrrolylcarbohydrazide (**1**) and previously synthesized in our laboratory α- and β-pyrrole aldehydes (**A**–**D**) [26] according to published procedures [39]. The aldehydes **A**–**D** (Table 1) were specifically chosen to introduce a second pyrrole heterocycle into the hydrazone molecules and explain some trends in the structure–activity relationship. The reactions were carried out in an acetic acid medium by heating equimolar amounts of both partners at the temperature of a boiling water bath for 30–180 min to complete the reaction (under TLC control until no presence of the starting hydrazide). Thus, four new hydrazones were prepared with yields in the range of 81–87%. The structures of hydrazones (**1A**–**D**) were identified through IR, ^1^H-NMR, ^13^C-NMR, and Mass spectra (see Supplementary Materials) and their purity was monitored by HPLC and melting point.

In the IR spectra, the stretching vibration of the ester group was found in the pyrrole ring within the range of 1698–1681 cm^−1^. IR absorption bands were detected for –NH– stretching vibrations within the range of 3440–3218 cm^−1^. In the ^1^H-NMR spectra of hydrazones (**1A**–**D**), the signals of the pyrrole ester groups –OCH_2_CH_3_ appeared in the form of a multiplet at 1.15–1.34 ppm for the –CH_3_ group and a multiplet at 4.12–4.30 ppm for the –CH_2_– group. The signals of the –CH_3_ groups attached to the pyrrole ring were detected as double singlets or as multiplets at 1.75–2.80 ppm. As multiplets were detected, signals for the –CH_2_– group attached to C_6_H_5_ (at 3.00–3.61 ppm), the C_6_H_4_ (at 6.32–7.01 ppm), and the C_6_H_5_ (at 6.99–7.35 ppm). The characteristic signals for the –CH– group attached to the pyrrole N-atom were visible as double dublets or as multiplets at 4.86–5.87 ppm. The signals of the –H on the 4-position in the pyrrole ring were detected as singlets at 6.20–6.48 ppm. In the obtained ^1^H-NMR spectra were detected the signals for the CH=N proton (at 7.85–8.60 ppm), the –NH-pyrrole (at 8.60–11.23 ppm), and the CONH (at 9.19–11.64 ppm).

### 2.2. Biological Study

#### 2.2.1. Safety Test (Cytotoxicity/Phototoxicity)

**Figure 4:**
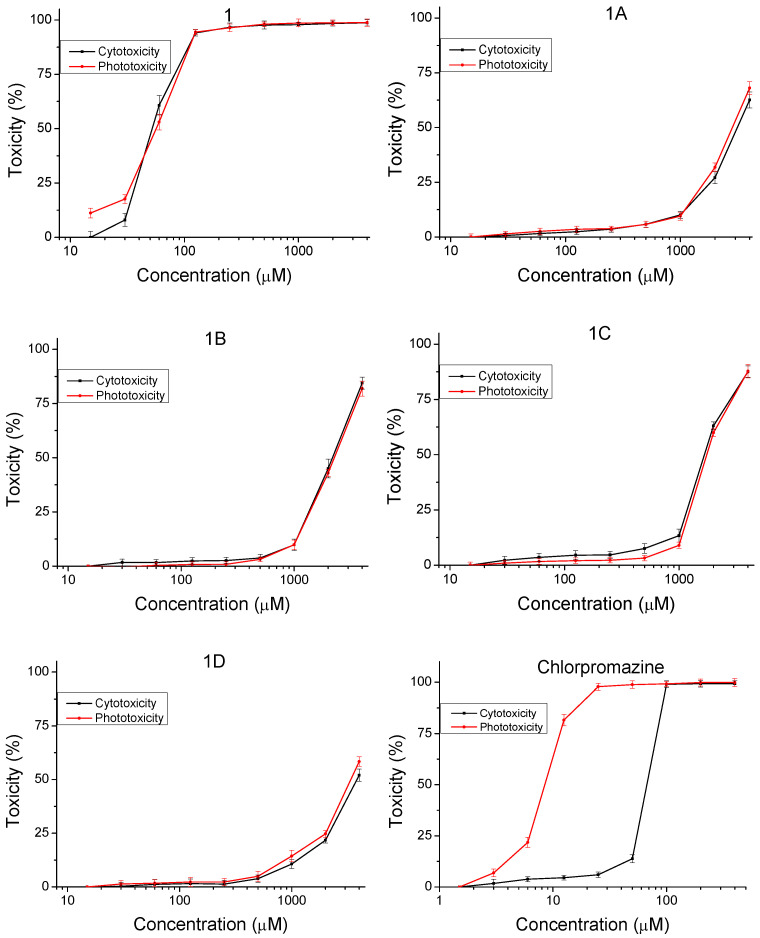
Cytotoxicity/phototoxicity of the studied compounds (**1**, **1A**–**D**) determined in the cell line BALB 3T3 clone A31. Chlorpromazine—positive control, *n* = 6.

The observed effect (cytotoxicity/phototoxicity) was of a dose-dependent type (Figure 4). From the obtained sigmoidal curves, the average CC_50_ values for the studied compounds were calculated (Table 2). The tested substances showed no phototoxicity (PIF < 2) and are generally safe with CC_50_ values > 1500 µM, except for compound **1**, which has a CC_50_ of 52.29 ± 2.63 µM. As a positive control, we used the phototoxic drug Chlorpromazine, where PIF = 7.21.

#### 2.2.2. Antiproliferative Activity Assay

**Figure 5:**
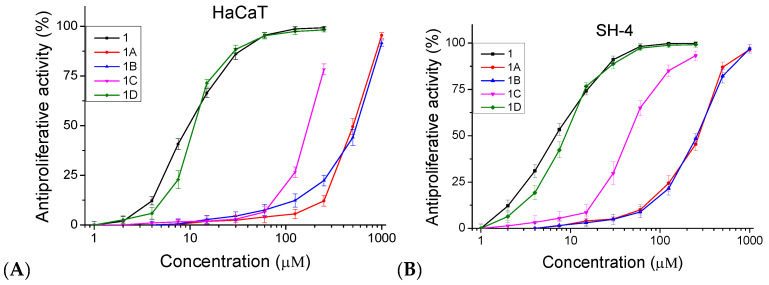
Antiproliferative effect of the investigated substances in (**A**) the non-tumorigenic cell line HaCaT and (**B**) melanoma cell line SH-4, *n* = 6.

The antiproliferative activity of the investigated substances was determined in non-tumor keratinocytes HaCaT (healthy tissue model) and SH-4 tumor cells (Figure 5A,B). The resulting sigmoidal curves show a dose-dependent effect. Compounds **1** and **1D** exhibited significant antiproliferative activity in non-tumor cells (HaCaT) with IC_50_ = 9.64 ± 0.58 and 11.03 ± 0.42, respectively (Table 3). A similar antiproliferative activity was also observed in tumor cells (SH-4). Keratinocytes are more resistant to the investigated compounds compared to tumor cells (for all substances SI > 1). The highest selective indices are observed for **1B** and **1C**, 2.11 and 3.83, respectively. In contrast, for the positive control (Cisplatin), the selectivity index is very low (SI = 0.38).

#### 2.2.3. Annexin V-FITC/PI Flow Cytometry Assay

**Figure 6:**
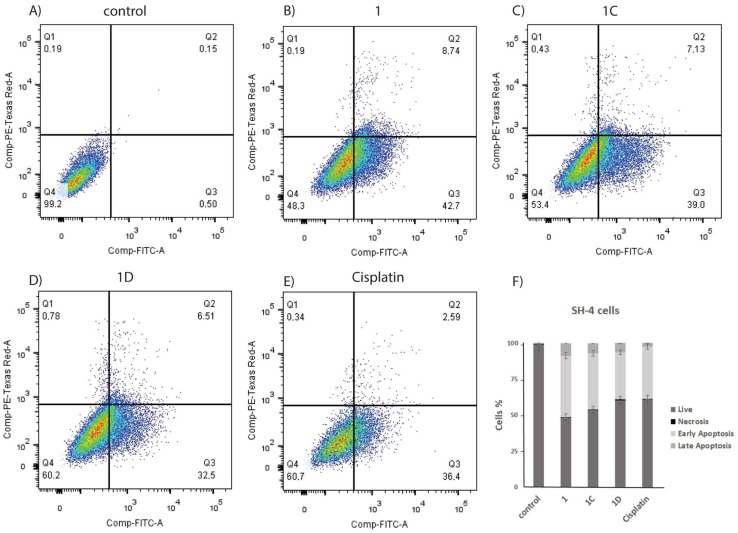
Effect of new pyrrole hydrazones on human cancer cell line SH-4 following 48 h treatment. (**A**–**D**) SH-4 cells treated with 6 µM parental compound **1** and its derivatives—**1C** (40 µM) and **1D** (8 µM) were subjected to both Annexin V-FITC and propidium iodide prior to analysis using a flow cytometer. (**E**) Cisplatin (20 µM) was used as a positive control. Dot plots representing control and treated cells. (**F**) Scatter plot represents the percentages of necrosis (upper left), late apoptosis (upper right), viable cells (lower left), and early apoptosis (lower right) populations.

In order to verify whether new compounds are able to induce apoptosis in SH-4 cells, we utilized FITC-Annexin V/PI staining and estimated the percentage of apoptotic cells by flow cytometry. The results showed that parental compound **1** treatment of SH-4 carcinoma cells significantly increased total apoptotic cell populations after 48 h incubation (Figure 6 and Supplementary Table S1) by 50% compared to the control group. We also noted that the β-aldehyde derivative with the highest selective index **1C** demonstrates greater induction of early apoptosis than other β-aldehyde derivative **1D**. The population fraction of total apoptotic cells was recorded to be around 46% after the treatment with 40 µM for 48 h compound **1C** and 39% for **1D** after the treatment with 8 µM. Our results also demonstrate that α-aldehyde derivative **1B** has 14% higher apoptotic potential than α-aldehyde derivative **1A**. We also showed that compound **1A** has comparable apoptotic activity with positive control—Cisplatin (see Supplementary Materials, Figure S26).

#### 2.2.4. Cell Cycle Analysis

**Figure 7:**
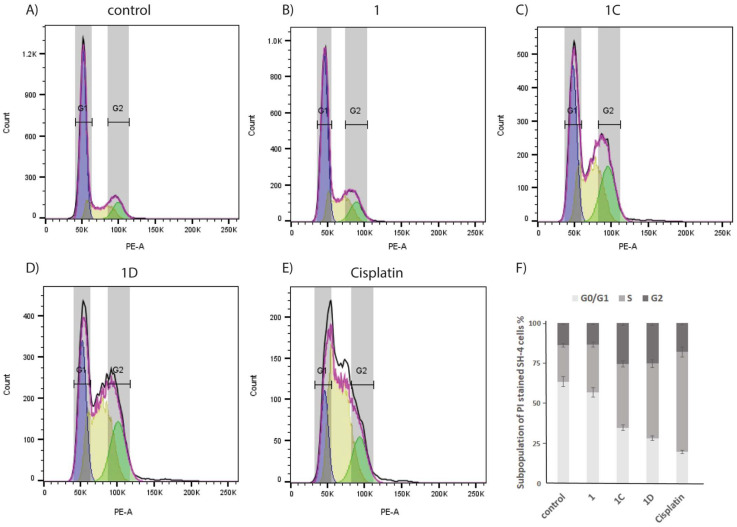
Effect of new pyrrole hydrazones on cell cycle distribution on human cancer cell line SH-4 following 48 h treatment. (**A**–**E**) SH-4 cells untreated (control) or treated with 6 µM parental compound **1** and its derivatives—**1C** (40 µM) and **1D** (8 µM). After treatment, cells were stained with PI and DNA content was analyzed using flow cytometry. A representative histogram is shown for each incubation condition. (**F**) Scatter plot represents the percentages of G1/G0, S, and G2 populations.

To examine how our pyrrole derivatives suppressed the growth of SH-4 cells, we performed flow cytometry analysis using the PI stain to evaluate DNA content. As shown in Figure 7 and Supplementary Table S2, our compounds with the strongest antiproliferative effect—**1C** and **1D** induced accumulation of cells mainly in the S phase, G2 phase, and a corresponding decrease in the G0/G1 phase. Treatment of SH-4 cells with β-aldehyde derivatives led to a significant elevation in the number of cells in S-phase from 21% in the control group to 38% for **1C** and 46% for **1D**, suggesting induction of S-phase arrest. When cells are exposed to stressors, checkpoint signal mechanisms are activated in the G1, S, or G2/M phase, resulting in cell-cycle arrest and often apoptosis [40], as we observed earlier in Figure 6. We also noted that β-aldehyde derivatives display a greater effect on cell cycle de-regulation when compared to their parental compound **1**. Our findings demonstrate that α-aldehyde derivative **1B** exhibits a slight increase in the S phase population, and no significant changes in the cell cycle distribution were detected for compound **1A** (see Supplementary Materials, Figure S27).

## 3. Discussion

The most commonly used method of treating oncological diseases is chemotherapy. It is applied alone, after surgical removal of the tumor, or in combination with other types of cancer therapy. Melanoma tumor cells are resistant to standard cytostatics, which makes the use of chemotherapy in melanoma ineffective [41,42]. To suppress tumor growth, it is necessary to apply high doses of cytostatic, which leads to the appearance of severe side effects and a significant reduction in the quality of life of cancer patients [43]. That is why studies on the use of new substances with antitumor activity in melanoma are relevant. Furthermore, antiproliferative activity, toxicity, and selectivity can be modified by incorporating pharmacologically active groups into the overall chemical structure of pyrrole hydrazones. In this study, we aimed to synthesize one main hydrazide (**1**) and four pyrrole hydrazones (**1A**–**D**). The hydrazones **1A** and **1B** were obtained by condensation of the main hydrazide (**1**) with α-pyrrole aldehydes (aldehydes **A** and **B**, see Table 1). The other two hydrazones, **1C** and **1D**, were synthesized using condensation of the same hydrazide with β-pyrrole aldehydes (aldehydes **C** and **D**, see Table 1). Phototoxicity is defined as a toxic response elicited by topically or systemically administered photoreactive chemicals after exposure to light. Phototoxic compounds contain heterocyclic structures, benzene nuclei, pyrrole derivatives, and other chemical groups with conjugated chemical bonds. The addition of various chemical groups to potentially phototoxic substances can influence their phototoxicity [44]. In medicine, a large number of phototoxic substances are used for the treatment of oncological diseases (5-Fluorouracil, Vinblastine, Vemurafenib, Paclitaxel, Doxorubicin, etc.) [45,46]. To avoid the negative side effects associated with the phototoxicity of these drugs, patients should not be exposed to direct sunlight. In order to obtain information about possible side effects related to phototoxicity, we performed a test to determine the phototoxic potential and safety of the synthesized substances (**1**, **1A**–**D**). The safety test showed that all newly synthesized hydrazones have significantly lower toxicity than the main substance (**1**). In addition, no phototoxicity was observed with any of the substances tested. Literary data presented by Tzankova et al. and Ibrahim et al. show that usually, the derivatives of hydrazones are of significant toxicity (IC_50_ < 100 µM) [47,48].

Our new pyrrole hydrazones exhibit very good inhibitory activity with IC_50_ values comparable and even smaller than currently existing pyrrole-based anticancer agents like VEGFR-2 inhibitor compounds **55a**–**d** [49,50], mimetics of protein–protein interaction compounds **1**, **9a**–**i**, **10a**, **b** and others. They also displayed selectivity to human melanoma cells similar to other compounds [51]. Our findings demonstrate that compounds **1C** and **1D** (obtained by condensation with β-aldehydes) exhibit greater antiproliferative activity than compounds **1A** and **1B** (obtained by condensation with α-aldehydes). It is likely that substitution in 3-position in general in the pyrrole ring (β-position) increases compound functionality by specific ligand–target cell protein recognition and interaction, but further SAR (structure–activity relationship) analysis is needed to recognize specific targets.

In particular, compound **1C** was the most selective pyrrole derivate among the tested compounds (SI = 3.83) against tumor cells. In SH-4 melanoma cells, the mean IC_50_ value was 44.63 ± 3.51 µM, which is comparable to standard cytostatics used in chemotherapy (Carboplatin IC_50_ = 18.2 µM, Temozolomide IC_50_ = 50 µM, Ribociclib IC_50_ = 34.96 µM, etc.) [52,53]. In our study, we observed that β-aldehyde derivatives **1C** and **1D** induce apoptosis and trigger S-phase cell cycle arrest. Taking into account the obtained new molecules presented herein reveals a very good impact according to their safety and biological activity. However, further studies are needed to understand the mechanism of action of the compounds based on the interaction with proteins that are involved in specific molecular cell pathways. Further studies are also needed to test the efficacy of **1C** in combination with chemotherapies commonly used in the treatment of melanoma. In our opinion, the results could justify further studies and preclinical analyses in mice to investigate the properties of the new pyrrole hydrazones and their potential application, at least as an adjuvant in the treatment of human melanoma.

## 4. Materials and Methods

The needed reagents for the synthesis of pyrrole derivatives ethyl alcohol (CAS Number: 64-17-5), chloroform (CAS Number: 67-66-3), hexane (CAS Number: 110-54-3), and glacial acetic acid (CAS Number: 64-19-7) are purchased from Valerus (Sofia, Bulgaria). The ethyl acetoacetate (CAS Number: 141-97-9) and thionyl chloride (CAS Number: 7719-09-7) are from Sigma-Aldrich (Ansbach, Germany). The synthetic reactions of the compounds were controlled by Thin Layer Chromatography (TLC) on aluminum plates covered with Silica gel 60 F254 (Merck, Darmstadt, Germany), using CHCl_3_/C_2_H_5_OH as a mobile phase. Yields were calculated for purified products. All reagents and solvents were used without any preliminary treatment. The melting points were measured in a capillary digital melting point apparatus IA 9200 (Electrothermal, London, UK) and were not corrected. IR spectra were measured using KBr tablets on a Varian 660 IR FT-IR Spectrometer (Agilent Technologies, Santa Clara, CA, USA). All ^1^H-NMR and ^13^C-NMR spectra were recorded in the appropriate solvent on a Bruker NEO 400 spectrometer (Bruker, Faelanden, Switzerland). Chemical shifts were expressed to tetramethylsilane (TMS) and were represented in (ppm). The mass spectrometry was recorded on the Shimadzu LC-MS/MS 8045 system (Shimadzu Corporation, Tokyo, Japan), with Agilent Poroshell 120 (CA, USA), 100 mm × 4.6 mm column. The mobile phase rate was 0.30 mL/min, and the column temperature was 40 °C. The following gradient elution was used: Mobile phase A: H_2_O (10% AcCN; 0.1% HCOOH); Mobile phase B: AcCN (5% H_2_O, 0.1% HCOOH). The gradient of the mobile phase starts with 80%A/20%B, passes through 5%A/95%B in 15 min, and returns to 80%A/20%B in 22 min. The Mass Spectrometry detector was used in SCAN/ESI+ mode of ionization with 3 L/min of the nebulizing gas flow, 10 L/min of the heating and drying gas flow, 350 °C interface temperature, 200 °C DL temperature, and 400 °C heat block temperature.

### 4.1. Synthesis

#### 4.1.1. Synthesis of Intermediate Ethyl Ester of the *N*-Pyrrolylcarboxylic Acid

0.04 mol SOCl_2_ was added dropwise at 0 °C to 30 mL dry ethanol under intensive stirring. Thereafter, 0.01 mol of the *N*-pyrrolylcarboxylic acid was added immediately, and the mixture was refluxed for 5 h to complete the reaction (TLC control). The solvent was removed under reduced pressure, and the resulting oil was dissolved in chloroform and washed successively with 1% solution of NaOH and water. The organic layer was dried over Na_2_SO_4_ and evaporated under reduced pressure. The residue consisted of the relevant ethyl ester, which was used in the next step without isolation and additional purification.

#### 4.1.2. Synthesis of *N*-Pyrrolylcarbohydrazide (**1**)

Here, 0.01 mol of the ethyl ester and 0.04 mol hydrazine hydrate (100%) were dissolved in 15 mL ethanol (99.7%). The mixture was refluxed for 8 h to complete the reaction under TLC control (the relevant mobile phase and Rf value are specified below). After cooling the ethanol solution, the product was isolated as a white solid and was recrystallized from ethanol.

#### 4.1.3. Synthesis of Hydrazones (**1A**–**D**)

0.01 mol of *N*-pyrrolylcarbohydrazide (**1**) and 0.01 mol of either of the relevant pyrrole aldehydes diethyl 5-formyl-3-methyl-1H-pyrrole-2,4-dicarboxylate (**A**), ethyl 5-formyl-2,4-dimethyl-1H-pyrrole-3-carboxylate (**B**), ethyl 4-formyl-3,5-dimethyl-1H- pyrrole-2-carboxylate (**C**) or ethyl 4-formyl-2,5-dimethyl-1H-pyrrole-3-carboxylate (**D**) (Table 1) were dissolved in 30 mL glacial acetic acid. The mixture was stirred at the temperature of a boiling water bath for 30–180 min to complete the reaction (under TLC control; the relevant mobile phase and Rf values are specified below). The products were precipitated after adding water. Hydrazones **1A** and **1B** were recrystallized from ethanol/water (ratio 10:1), and the other two hydrazones, **1C** and **1D**, from ethanol.

Physical characteristics and structure elucidation of the new compounds (chemical name; m.p. (°C); yield (%); Rf (at the given ratio CHCl_3_:C_2_H_5_OH = 10:0.4 at 20 ± 1 °C); IR in KBr, [cm^−1^]; ^1^H-NMR and ^13^C-NMR (400 MHz, solvent, ppm); HPLC-MS:

*Ethyl-5-(4-chlorophenyl)-1-(1-hydrazinyl-1-oxo-3-phenylpropan-2-yl)-2-methyl-1H-pyrrole-3-carboxylate* (**1**): m.p. 170–173 °C; yield 78%; Rf 0.60; IR: 3305, 3204 (NH), 2978 (CH_3_), 2870 (CH_2_), 1686 (C=O), 1637 (Amide I), 1571 (Amide II), 1241 (C-O), 820 (p-disubstituted C_6_H_4_). ^1^H-NMR (CDCl_3_): 1.26 (t, J = 7.1 Hz, 3 H), 2.57 (s, 3 H), 3.04–3.10 (m, 1 H), 3.52–3.57 (m, 3 H), 4.18–4.24 (q, J = 7.1 Hz, 2 H), 4.70–4.74 (m, 1 H), 6.29 (s, 1 H), 6.32–6.43 (m, 2 H), 6.63–6.66 (m, 2 H), 7.06–7.19 (m, 6 H). ^13^C-NMR (CDCl_3_): 13.4 (1C, C(14)), 14.5 (1C, C(17)), 36.1 (1C, C(11)), 59.8 (1C, C(16)), 60.3 (1C, C(8)), 110.7 (1C, C(9)), 114.6 (1C, C(12)), 127.0 (3C, C(2), C(6), C(4′)), 128.5 (2C, C(2′), C(6′)), 128.7 (2C, C(3′), C(5′)), 129.1 (2C, C(3), C(5)), 129.9 (1C, C(1)), 131.0 (1C, C(4)), 134.3 (1C, C(7)), 135.7 (1C, C(1′)), 136.6 (1C, C(10)), 165.1 (1C, C(15)), 170.4 (1C, C(13)). HPLC-MS: Mm_exact_ [g/mol] calcul. 425.15, [M+nH]^+^ observed 426.05; t_R_ [min] 9.272; Chromatographic purity: 95%.
*Diethyl(E)-5-((2-(2-(5-(4-chlorophenyl)-3-(ethoxycarbonyl)-2-methyl-1H-pyrrol-1-yl)-3-phenylpropanoyl)hydrazineylidene)methyl)-3-methyl-1H-pyrrole-2,4-dicarboxylate* (**1A**): m.p. 109–112 °C; yield 87%; Rf 0.44; IR: 3440 (NH), 2979 (CH_3_), 2871 (CH_2_), 1698 (C=O), 1603 (Amide I), 1527 (Amide II), 1243 (C-O), 818 (p-disubstituted C_6_H_4_). ^1^H-NMR (CDCl_3_): 1.21–1.34 (m, 9H), 2.43 (ds, 3H), 2.62 (ds, 3H), 3.07–3.09 (m, 1H), 3.42–3.61 (m, 1H), 4.16–4.30 (m, 6H), 4.86–4.90 (m, 1H), 6.48 (s, 1H), 6.59–6.82 (m, 4H), 6.99–7.19 (m, 5H), 8.60 (brs, 1H), 9.06 (brs, 1H), 10.54 (brs, 1H). ^13^C-NMR (CDCl_3_): 11.8 (1C, C(26)), 14.3 (1C, C(14)), 14.3–14.4 (3C, C(17), C(25), C(29)), 36.3 (1C, C(11)), 59.9–60.8 (3C, C(16), C(24), C(28)), 117.7 (1C, C(8)), 122.4 (1C, C(9)), 128.5 (2C, C(22), C(12)), 128.6 (3C, C(2), C(6), C(4′)), 128.7 (5C, C(20), C(2′), C(3′), C(5′), C(6′)), 129.0 (3C, C(3), C(5), C(21)), 130.9 (3C, C(19), C(1), C(4)), 136.2 (2C, C(7), C(1′)), 140.0 (1C, C(18)), 160.7 (1C, C(10)), 165.0 (1C, C(27)), 165.1 (2C, C(15), C(23)), 166.6 (1C, C(13)). HPLC-MS: Mm_exact_ [g/mol] calcul. 660.24, [M+nH]^+^ observed 661.20, [M+Na]^+^ observed 683.15; t_R_ [min] 13.782; Chromatographic purity: 95%.
*Ethyl(E)-5-(4-chlorophenyl)-1-(1-(2-((4-(ethoxycarbonyl)-3,5-dimethyl-1H-pyrrol-2-yl)methylene)hydrazineyl)-1-oxo-3-phenylpropan-2-yl)-2-methyl-1H-pyrrole-3-carboxylate* (**1B**): m.p. 129–132 °C; yield 84%; Rf 0.70; IR: 3420 (NH), 2978 (CH_3_), 2930 (CH_2_), 1698 (C=O), 1614 (Amide I), 1526 (Amide II), 1244 (C-O), 819 (p-disubstituted C_6_H_4_). ^1^H-NMR (CDCl_3_): 1.21–1.30 (m, 6H), 1.98–2.56 (m, 9H), 3.00–3.06 (m, 1H), 3.40–3.57 (m, 1H), 4.17–4.21 (m, 4H), 4.86–4.90 (m, 1H), 6.28 (s, 1H), 6.32–7.01 (m, 4H), 7.04–7.19 (m, 5H), 7.85 (s, 1H), 8.93 (brs, 1H), 10.13 (brs, 1H). ^13^C-NMR (CDCl_3_): 10.9 (1C, C(26)), 14.2 (1C, C(14)), 14.4 (1C, C(27)), 14.5 (2C, C(17), C(25)), 36.1 (1C, C(11)), 59.4–60.5 (2C, C(16), C(24)), 112.0 (1C, C(8)), 121.8 (1C, C(9)), 127.0 (2C, C(21), C(12)), 128.5 (1C, C(22)), 128.6 (3C, C(2), C(6), C(4′)), 128.7 (2C, C(2′), C(6′)), 128.9 (2C, C(3′), C(5′)), 129.2 (2C, C(3), C(5)), 130.8 (3C, C(19), C(1), C(4)), 134.5 (1C, C(1′)), 136.3 (2C, C(7), C(20)), 138.5 (1C, C(18)), 141.0 (1C, C(10)), 165.2 (2C, C(15), C(23)), 165.4 (1C, C(13)). HPLC-MS: Mm_exact_ [g/mol] calcul. 602.23, [M+nH]^+^ observed 603.05, [M+Na]^+^ observed 625.20; t_R_ [min] 13.002; Chromatographic purity: 95%.
*Ethyl(E)-4-((2-(2-(5-(4-chlorophenyl)-3-(ethoxycarbonyl)-2-methyl-1H-pyrrol-1-yl)-3-phenylpropanoyl)hydrazineylidene)methyl)-3,5-dimethyl-1H-pyrrole-2-carboxylate* (**1C**): m.p. > 230 °C; yield 81%; Rf 0.63; IR: 3218 (NH), 2977 (CH_3_), 1694 (C=O), 1608 (Amide I), 1568 (Amide II), 1244 (C-O), 822 (p-disubstituted C_6_H_4_). ^1^H-NMR (DMSO-d_6_): 1.20–1.30 (m, 6 H), 2.05, 2.10 (ds, 3 H), 2.37–2.57 (m, 6 H), 3.31–3.45 (m, 2 H), 4.12–4.24 (m, 4 H), 5.03, 5.05 and 5.85, 5.87 (dd, 1 H), 6.20 (s, 1 H), 6.70–6.90 (m, 4 H), 7.07–7.35 (m, 5 H), 8.06, 8.38 (ds, 1 H), 11.02, 11.23 (ds, 1 H), 11.64, 11.69 (ds, 1 H). ^13^C-NMR (DMSO-d_6_): 11.2 (1C, C(26)), 12.1 (1C, C(14)), 12.7 (1C, C(27)), 14.8 (2C, C(17), C(25)), 39.3 (1C, C(11)), 59.3–59.8 (2C, C(16), C(24)), 115.6 (1C, C(8)), 116.0 (1C, C(9)), 118.1 (1C, C(21)), 127.0 (1C, C(12)), 127.1 (1C, C(22)), 127.4 (3C, C(2), C(6), C(4′)), 128.5–128.6 (2C, C(2′), C(6′)), 128.7–128.8 (2C, C(3′), C(5′)), 129.2 (2C, C(3), C(5)), 131.7 (1C, C(19)), 132.9 (1C, C(1)), 133.1 (1C, C(4)), 134.0 (1C, C(1′)), 135.6 (1C, C(7)), 136.0 (1C, C(20)), 136.9 (1C, C(18)), 142.2 (1C, C(10)), 161.1 (1C, C(15)), 164.9 (1C, C(23)), 169.6 (1C, C(13)). HPLC-MS: Mm_exact_ [g/mol] calcul. 602.23, [M+nH]^+^ observed 603.10, [M+Na]^+^ observed 625.20; t_R_ [min] 12.395; Chromatographic purity: 95%.
*Ethyl(E)-5-(4-chlorophenyl)-1-(1-(2-((4-(ethoxycarbonyl)-2,5-dimethyl-1H-pyrrol-3-yl)methylene)hydrazineyl)-1-oxo-3-phenylpropan-2-yl)-2-methyl-1H-pyrrole-3-carboxylate* (**1D**): m.p. > 230 °C; yield 82%; Rf 0.45; IR: 3331 (NH), 2980 (CH_3_), 2906 (CH_2_), 1681 (C=O), 1607 (Amide I), 1541 (Amide II), 1247 (C-O), 817 (p-disubstituted C_6_H_4_). ^1^H-NMR (CDCl_3_): 1.15–1.34 (m, 6H), 1.75–1.99 (m, 3H), 2.36–2.42 (m, 3H), 2.62–2.80 (m, 3H), 3.04–3.31 (m, 2H), 4.18–4.26 (m, 4H), 4.88, 4.90 and 5.67, 5.69 (dd, 1H), 6.27 (ds, 1H), 6.53–6.71 (m, 4H), 6.99–7.19 (m, 5H), 8.37 (s, 1H), 8.60 (m, 1H), 9.19 (m, 1H). ^13^C-NMR (CDCl_3_): 14.4 (2C, C(17), C(25)), 14.5 (3C, C(26), C(14), C(27)), 37.5 (1C, C(11)), 59.8 (2C, C(16), C(24)), 110.0 (1C, C(8)), 126.9 (2C, C(9), C(21)), 128.2 (2C, C(12), C(22)), 128.3 (3C, C(2), C(6), C(4′)), 128.5 (4C, C(2′), C(6′), C(3′), C(5′)), 128.6 (2C, C(3), C(5)), 128.8 (1C, C(19)), 129.0 (2C, C(1), C(4)), 131.0 (3C, C(1′), C(7), C(20)), 131.5 (1C, C(18)), 134.1 (1C, C(10)), 165.1 (1C, C(15)), 165.9 (1C, C(23)), 170.0 (1C, C(13)). HPLC-MS: Mm_exact_ [g/mol] calcul. 602.23, [M+nH]^+^ observed 603.10, [M+Na]^+^ observed 625.15; t_R_ [min] 10.198; Chromatographic purity: 98%.

### 4.2. Biological Assay

#### 4.2.1. Cell Culture and Reagents

The human keratinocytes HaCaT (CLS, cat. № 300493) were obtained from CLS Cell Lines Service GmbH (CLS, Eppelheim, Germany). The human melanoma SH-4 (ATCC^®^ CRL-7724TM) and mouse embryonic fibroblasts BALB/3T3 clone A31 (ATCC^®^ CCL-163TM) were obtained from American Type Cultures Collection (ATCC, Manassas, VA, USA). Dulbecco’s modified Eagle’s medium (DMEM), fetal bovine serum (FBS), trypsin-EDTA solution, antibiotics (penicillin and streptomycin), Neutral Red and MTT-dye were purchased from Sigma-Aldrich, Schnelldorf, Germany. The disposable consumables were supplied by Orange Scientific, Braine-l’Alleud, Belgium. Cells were cultured in DMEM 4.5 g/l glucose, supplemented with 10% (*v*/*v*) FBS, 100 U/mL penicillin, and 0.1 mg/mL streptomycin in an incubator at 37 °C, 5% CO_2_ and 95% humidity. Plastic flasks of 75 cm^2^ were used to grow the cells.

#### 4.2.2. Safety Test

The Neutral Red Uptake in vitro assay is a colorimetric method for assessing cell viability [54]. This method is based on the ability of living cells to incorporate Neutral Red dye into lysosomes. Mouse embryonic fibroblasts were seeded at 1 × 10^4^ cells/100 μL culture medium/well in 96-well cell culture microplates. The cells were incubated for 24 h to allow cell adhesion. The cells were then treated with a solution of the test substances in double-increasing concentrations (from 15 to 4000 μM). The phototoxicity test was performed in parallel on a second 96-well plate [55], which was irradiated with a solar simulator (LED lamp—Helios-iO (SERIC Ltd., Tokyo, Japan) for 10 min (irradiation dose 0.86 J). After 24 h of incubation, a culture medium containing Neutral Red was added. After 3 h of incubation, the wells were washed with PBS, and a Desorb solution (50% Ethanol, 1% Acetic acid, 49% dH_2_O) was added. The optical density (OD) was measured by a microplate reader at λ = 540 nm. The percentage of cytotoxicity was calculated according to the following formula:

Cytotoxicity (%) = (1 − (OD_540_ (sample)/OD_540_ (negative control))) × 100

Phototoxicity is expressed by Photoirritation factor (PIF), PIF = CC_50_ − Irr/CC_50_ +Irr*

* Irradiation

#### 4.2.3. Antiproliferative Activity

The antiproliferative activity of the studied substances was determined by MTT dye reduction assay [56]. The assay is based on the metabolism of MTT by reductases in the mitochondria to formazan, the concentration of which can be determined photometrically. The measured absorbance is an indicator of cell viability and metabolic activity. Cells were seeded in 96-well cell culture plates (1 × 10^3^ cells/well). Followed by incubation at 37 °C, 5% CO_2_ for 24 h. Followed by incubation for 72 h of the cells with the studied substances in twice increasing concentrations. After washing with PBS, 100 µL/well of MTT solution (0.5 mg/mL) was added. After 3 h of incubation, the 96-well plates were washed with PBS, and 100 µL/well of lysing solution (DMSO/Ethanol = 1/1) was added. Optical density was measured at a wavelength of 540 nm. The antiproliferative activity expressed in % of the negative control was calculated according to the following formula:

Antiproliferative activity (%) = (1 − (OD_540_ (sample)/OD_540_ (negative control))) × 100

The IC_50_ values (a concentration that inhibited 50% cell proliferation) were calculated using non-linear regression analysis (GraphPad Prizm8 Software). The statistical analysis included the application of one-way ANOVA followed by Bonferroni’s post hoc test. *p* < 0.05 was accepted as the lowest level of statistical significance. All results are presented as mean ± SD.

#### 4.2.4. Annexin V-FITC/PI Flow Cytometry Assay

Apoptosis was discriminated with the annexin V-FITC/PI test according to the manufacturer’s protocol (SIGMA-ALDRICH APOAF). Briefly, the SH-4 cell line was cultured in a 6-well plate at a density of 2 × 10^5^ cells/well in 10% FBS-DMEM and treated with compounds **1**, **1A**, **1B**, **1C**, **1D** at their IC_50_ doses (respectively, 6 µM, 260 µM, 260 µM, 40 µM, and 8 µM) and cisplatin (20 µM) as positive control for 48 h. After completion of each drug incubation, cell media that may contain cells in suspension was centrifuged at 350× *g* for 5 min. Subsequently, the cell monolayers were washed once with PBS, and cells were detached with trypsin and then collected together with a centrifuged pellet. The cells were washed twice with cold PBS and then resuspended in a Binding Buffer (0.1 M Hepes/NaOH (pH 7.4), 1.4 M NaCl, 25 mM CaCl_2_) at a concentration of 1 × 10^5^ cells/mL. The cells were incubated with 5 µL of FITC Annexin V and 10 µL PI for 20 min at RT (25 °C) in the dark. The analysis was performed with BD FACS DIVA software version 7.0 (BD Biosciences, Franklin Lakes, NJ, USA). Data were analyzed as follows: lower left quadrant, viable cells (Annexin V−/PI−); lower right quadrant, early apoptotic cells (Annexin V+/PI−); upper right quadrant, late apoptotic cells (annexin V+/PI+); upper left quadrant, necrotic cells (annexin V−/PI+). Three independent experiments were performed, with two replicates per experiment.

#### 4.2.5. Cell Cycle Analysis

The SH-4 cells were cultured in a 12-well plate in 10% FBS-DMEM and treated with compounds **1**, **1A**, **1B**, **1C**, and **1D** at their IC_50_ doses (respectively 6 µM, 260 µM, 260 µM, 40 µM and 8 µM) and cisplatin (20 µM) as positive control for 48 h. After incubation, cells were washed twice with ice-cold PBS and fixed with ice-cold 70% ethanol at −20 °C overnight. The cells were treated with 20 µg/mL RNase A at 37 °C for 60 min, then washed with PBS and finally stained with 20 µg/mL PI in the dark for 30 min. The cellular DNA content for the cell cycle distribution analysis was performed with the system software (BD FACS DIVA software (BD Biosciences, Franklin Lakes, NJ, USA)), plotting at least 10,000 events per sample. Three independent experiments were performed, with two replicates per experiment.

## Figures and Tables

**Table 1:** Structure of pyrrole aldehydes **A**–**D**.

Aldehyde A	Aldehyde B	Aldehyde C	Aldehyde D
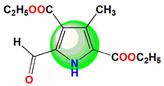	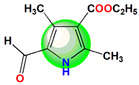	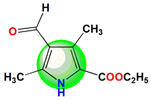	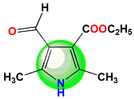

**Table 2:** Cytotoxicity/phototoxicity in BALB 3T3 cell line of the investigated compounds, mean CC_50_ values ± SD and PIF factor.

Compounds	Mean CC_50_ ± SD (μM)		PIF *
	−Irr	+Irr	
**1**	52.29 ± 2.63	55.83 ± 4.49	0.94
**1A**	3140.9 ± 167.34	2839.49 ± 123.89	1.11
**1B**	2182.68 ± 153.8	2265.47 ± 55.39	0.96
**1C**	1667.01 ± 41.52	1750.95 ± 31.68	0.95
**1D**	3799.84 ± 184.8	3374.23 ± 129.79	1.13
Chlorpromazine **	65.78 ± 2.64	9.13 ± 0.72	7.21

* Photo Irritation Factor: PIF < 2 not phototoxic, 2 < PIF < 5 probable phototoxicity, PIF > 5 phototoxic, ** Chlorpromazine (positive control).

**Table 3:** Mean IC_50_ values and selectivity index.

Compounds	Mean IC_50_ ± SD (μM)		SI *
	HaCaT	SH-4	
**1**	9.64 ± 0.58	6.86 ± 0.6	1.41
**1A**	503.11 ± 31.08	269.55 ± 13.53	1.87
**1B**	544.36 ± 26.8	258.27 ± 16.07	2.11
**1C**	171.12 ± 4.09	44.63 ± 3.51	3.83
**1D**	11.03 ± 0.42	8.72 ± 0.6	1.26
Cisplatin **	8.64 ± 0.42	22.78 ± 1.47	0.38

* SI—Selective index, SI = IC_50_ (non-tumor cell line)/IC_50_ (tumor cell line), ** Cisplatin (positive control).

## Data Availability

Data are contained within the article and Supplementary Materials.

## Supplementary Materials

The following supporting information can be downloaded at https://www.mdpi.com/article/10.3390/molecules29235499/s1; Figures S1–S5: IR spectra of targeted compounds; Figures S6–S10: ^1^H-NMR spectra of targeted compounds; Figures S11–S15: ^13^C-NMR spectra of targeted compounds; Figures S16–S25: HPLC-MS spectra of targeted compounds; Table S1: Percentage of cell populations displaying viable and apoptotic trends after treatment with new pyrrole hydrazones conducted via flow cytometry; Table S2: The percentage of cells in the G1, S and G2 phases of the cell cycle after treatment with new pyrrole hydrazones conducted via Flow Cytometry Assay; Figure S26. Effect of new pyrrole hydrazones on human cancer cell line SH4 following 48 h treatment. (A–D) SH-4 cells treated with 6 µM parental compound 1 and its derivatives—1A (260 µM) and 1B (260 µM) were subjected to both Annexin V-FITC and propidium iodide prior to analysis using a flow cytometer. (E) Cisplatin (20 µM) was used as a positive control. Dot plots representing control and treated cells. (F) Scatter plot represents the percentages of necrosis (upper left), late apoptosis (upper right), viable cells (lower left), and early apoptosis (lower right) populations; Figure S27. Effect of new pyrrole hydrazones on cell cycle distribution on human cancer cell line SH-4 following 48 h treatment. (A–D) SH-4 cells untreated (control) or treated with 6 µM parental compound 1 and its derivatives—1A (260 µM) and 1B (260 µM). After treatment, cells were stained with PI and DNA content was analyzed using flow cytometry. A representative histogram is shown for each incubation condition. (F) Scatter plot represents the percentages of G1/G0, S, and G2 populations.
