# Supplementary material for: Synthesis, Cytotoxicity and Antiproliferative Effect of New Pyrrole Hydrazones
Source: Molecules. 2024 Nov 21;29(23):5499. doi: 10.3390/molecules29235499 (PMC11643554; doi:10.3390/molecules29235499)
Supplement: Supplementary file 1 [file molecules-29-05499-s001.zip › molecules-3256897-supplementary.pdf]

Article

Synthesis, Cytotoxicity and Antiproliferative Effect of New Pyrrole Hydrazones

Stanislava Vladimirova [1,*], Rossitsa Hristova [2], Ivan Iliev [3]

1 Department of Organic Synthesis, University of Chemical Technology and Metallurgy, 8 Kliment Ohridski Blvd., 1756 Sofia, Bulgaria
2 Roumen Tsanev Institute of Molecular Biology, Bulgarian Academy of Sciences, 1113 Sofia, Bulgaria; rosi_hristova@hotmail.com
3 Institute of Experimental Morphology, Pathology and Anthropology with Museum, Bulgarian Academy of Sciences, 1113 Sofia, Bulgaria; taparsky@abv.bg
* Correspondence: vladimirova.s@uctm.edu

Contents

Figures S1-S5: IR spectra of targeted compounds page 2-4

Figures S6-S10: ^{1}H-NMR spectra of targeted compounds page 5-7

Figures S11-S15: ^{13}C-NMR spectra of targeted compounds page 8-10

Figures S16-S25: HPLC-MS spectra of targeted compounds page 11-15

Table S1: Percentage of cell populations displaying viable and apoptotic trends after treatment with new pyrrole hydrazones conducted via flow cytometry page 16

Figure S26. Effect of new pyrrole hydrazones on human cancer cell line SH4 following 48h treatment. A-D) SH-4 cells treated with *6μM* parental compound **1** and its derivatives – **1A** (260 μM) and **1B** (260 μM) were subjected to both Annexin V-FITC and propidium iodide prior to analysis using flow cytometer. E) Cisplatin (20 μM) was used as a positive control. Dot plots representing control and treated cells. F) Scatter plot represents the percentages of necrosis (upper left), late apoptosis (upper right), viable cells (lower left), and early apoptosis (lower right) populations page 16

Table S2: The percentage of cells in the G1, S and G2 phases of the cell cycle after treatment with new pyrrole hydrazones conducted via Flow Cytometry Assay. page 17

Figure S27. Effect of new pyrrole hydrazones on cell cycle distribution on human cancer cell line SH-4 following 48h treatment. A-D) SH-4 cells untreated (control) or treated with *6μM* parental compound **1** and its derivatives **– 1A (**260μM) and **1B** (260 μM). After treatment, cells were stained with PI and DNA content analyzed by flow cytometry. A representative histogram is shown for each incubation condition. F) Scatter plot represents the percentages of G1/G0, S and G2 populations. page 17

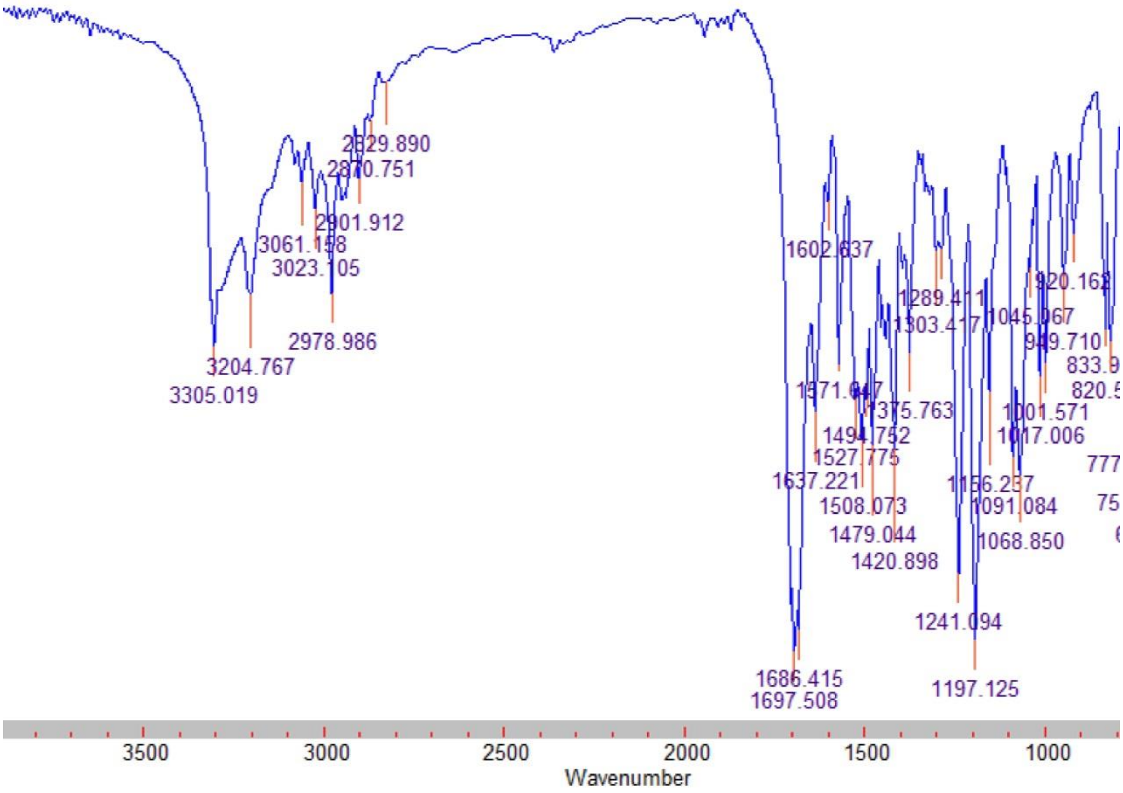

Figure S1. IR spectrum of compound 1

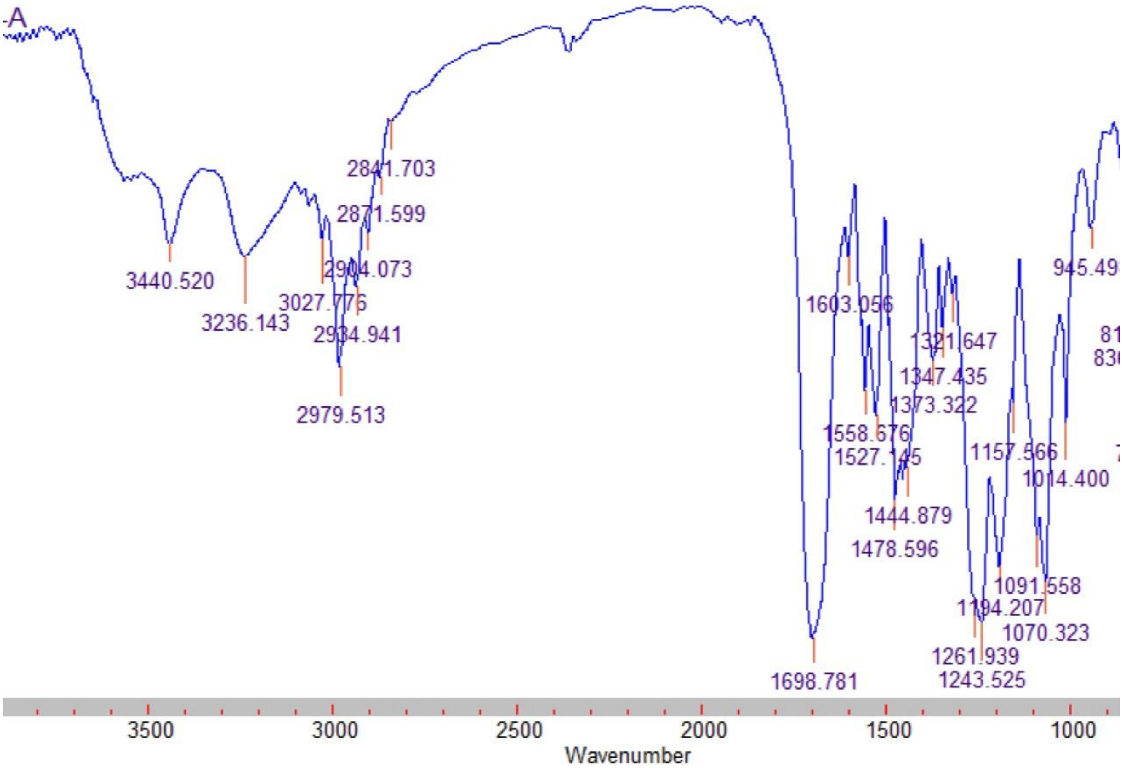

Figure S2. IR spectrum of compound 1A

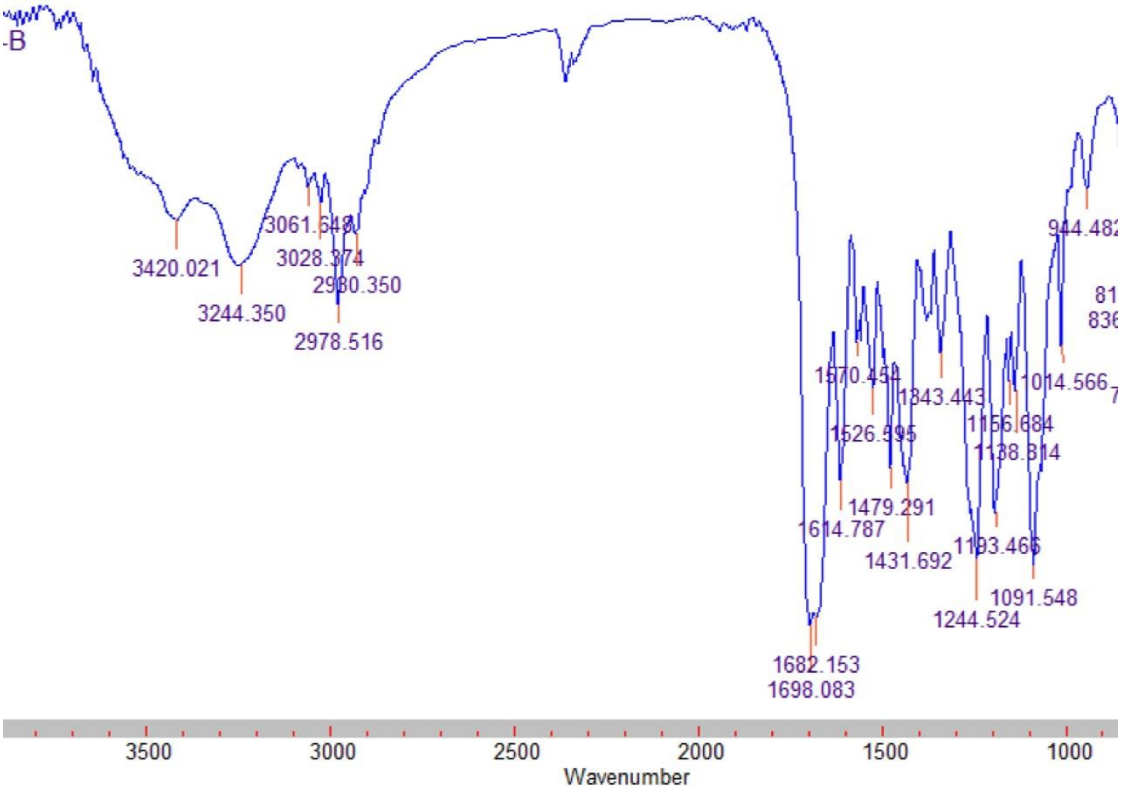

Figure S3. IR spectrum of compound 1B

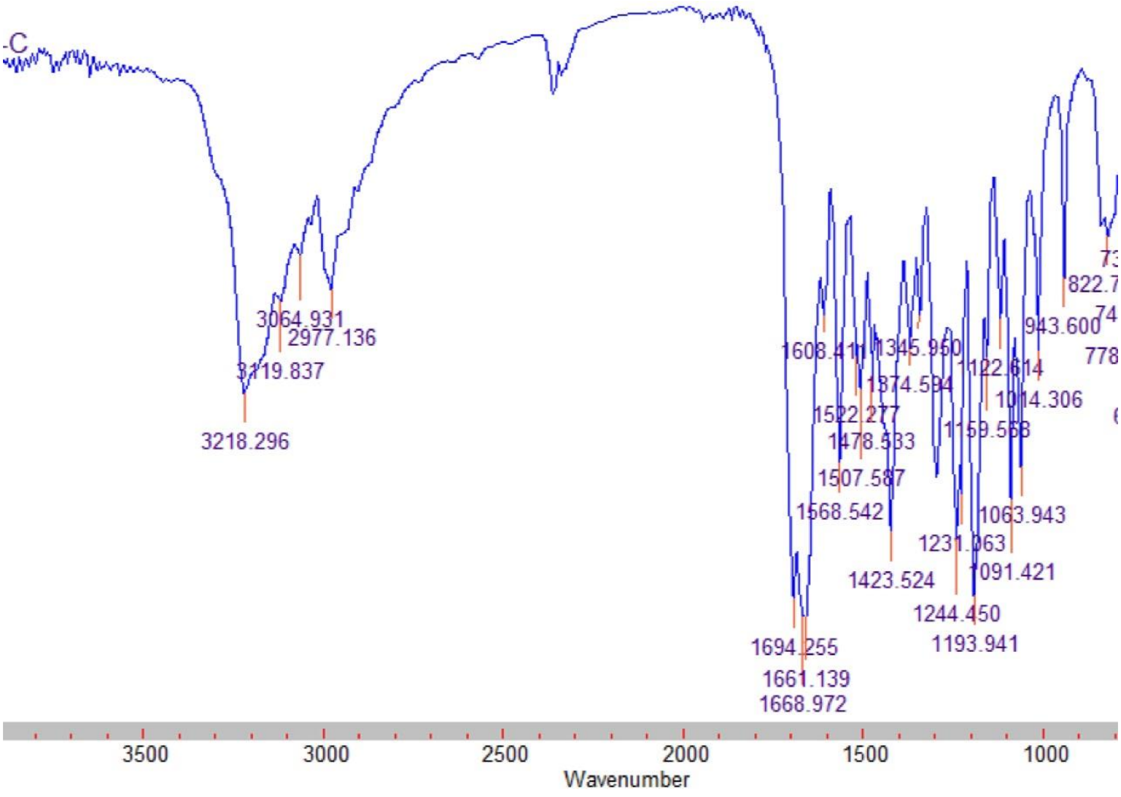

Figure S4. IR spectrum of compound 1C

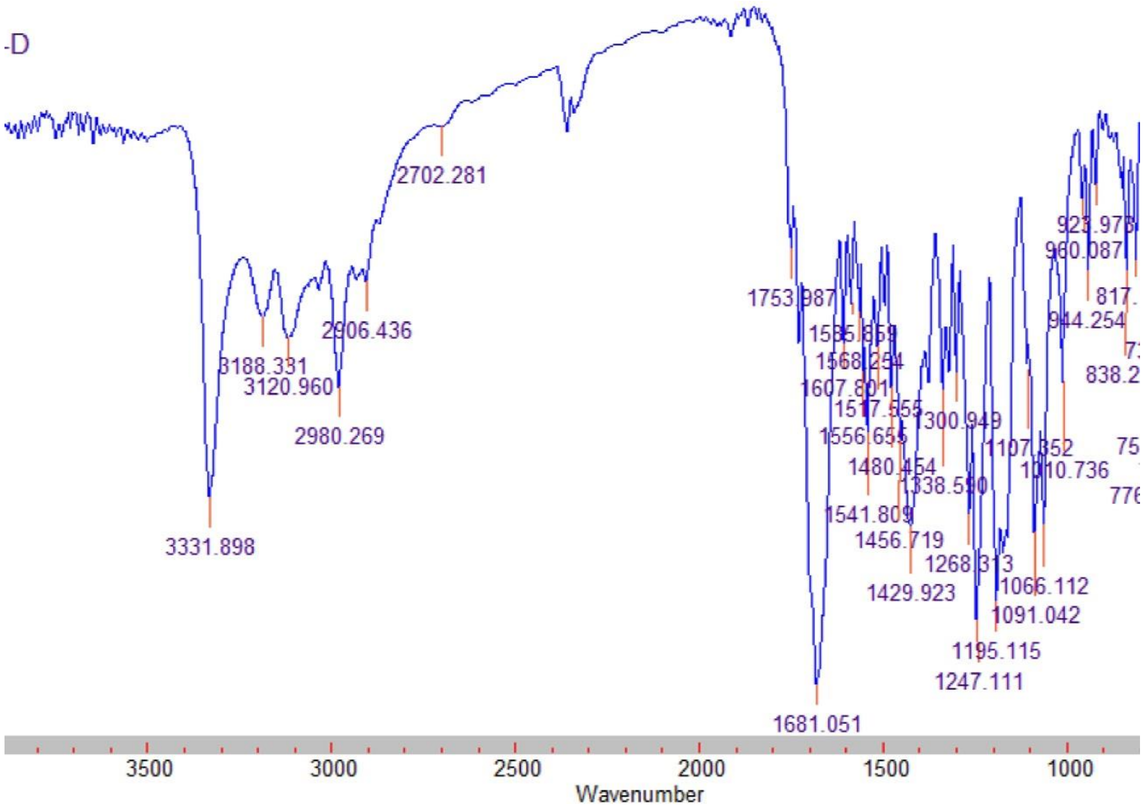

Figure S5. IR spectrum of compound 1D

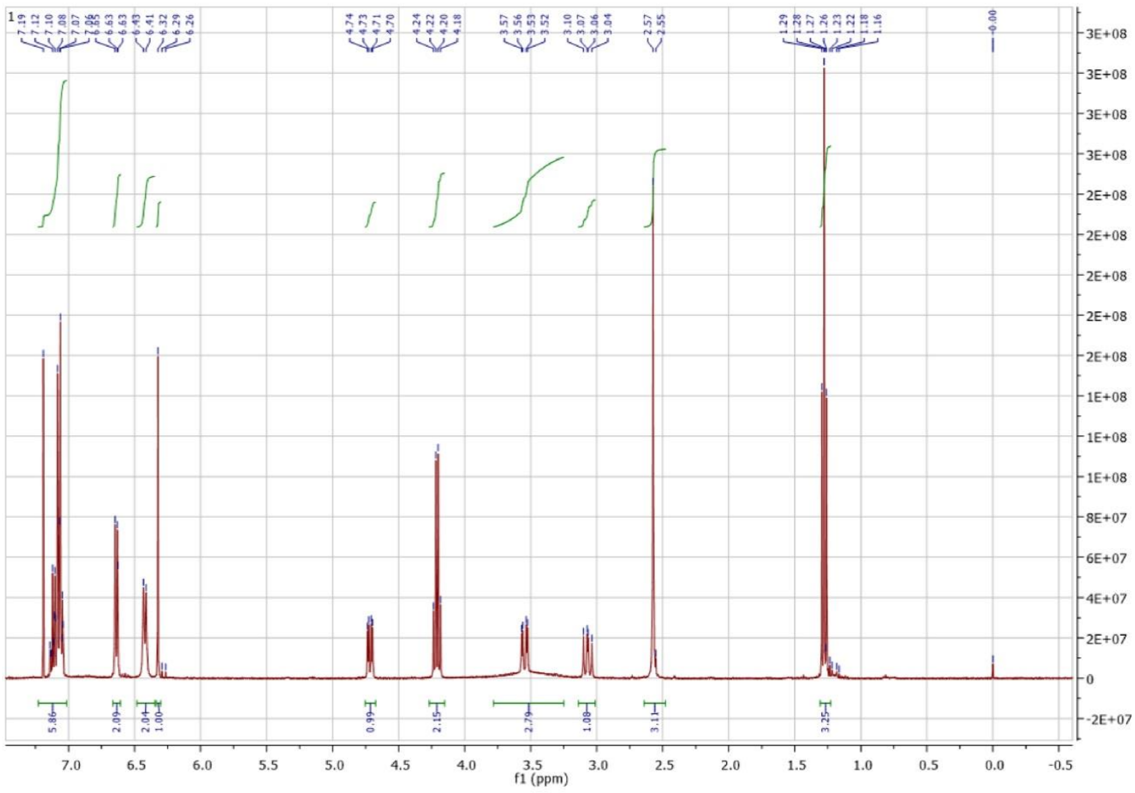

Figure S6. **^{1}H-NMR spectrum of compound 1**

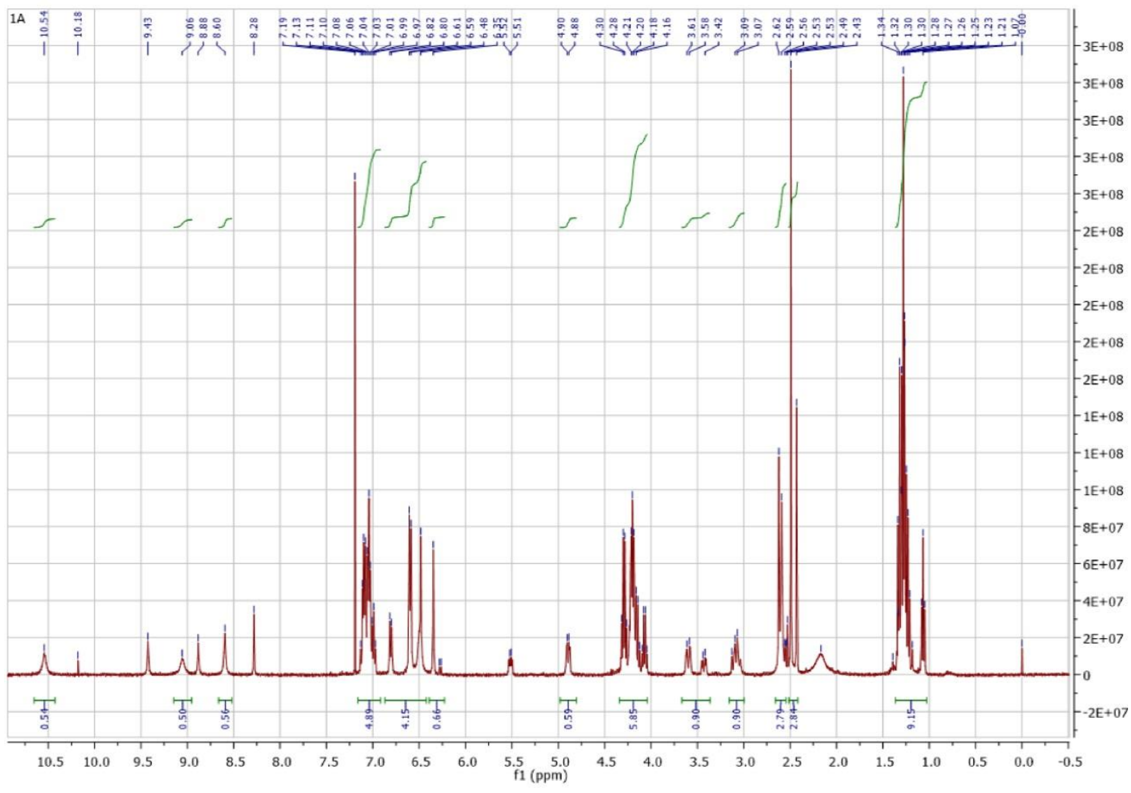

Figure S7. **^{1}H-NMR spectrum of compound 1A**

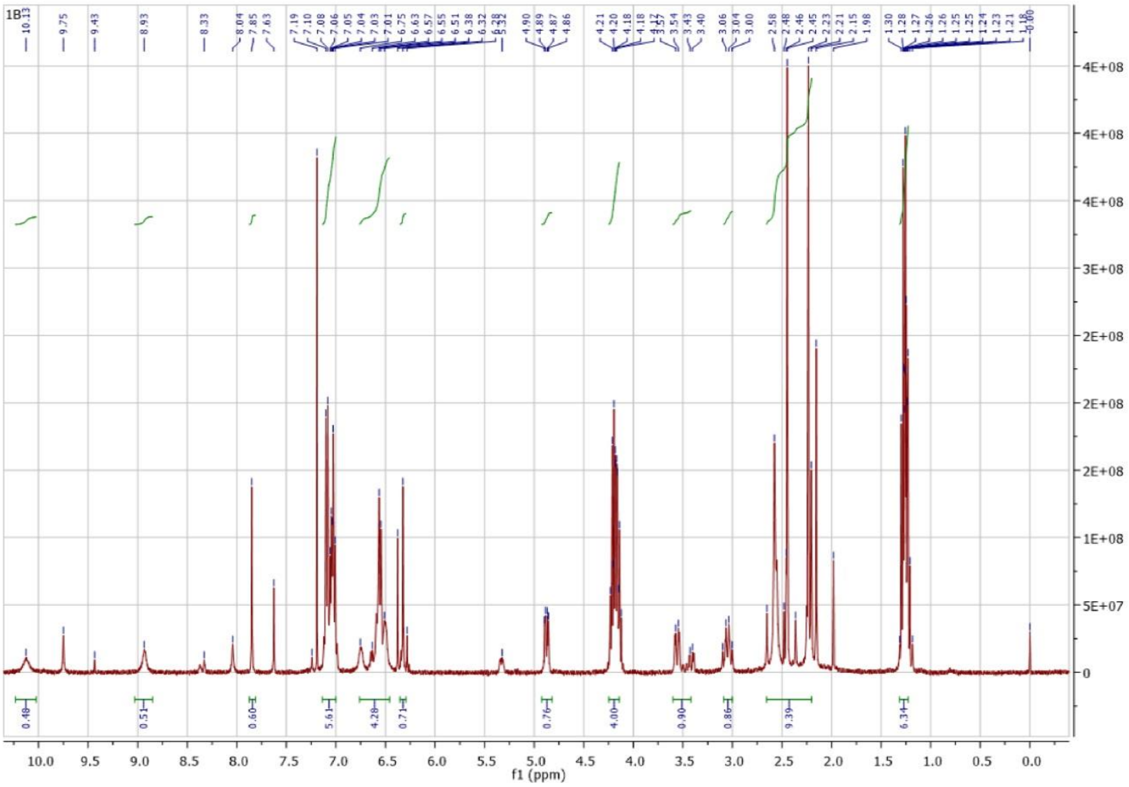

Figure S8. ^{1}H-NMR spectrum of compound 1B

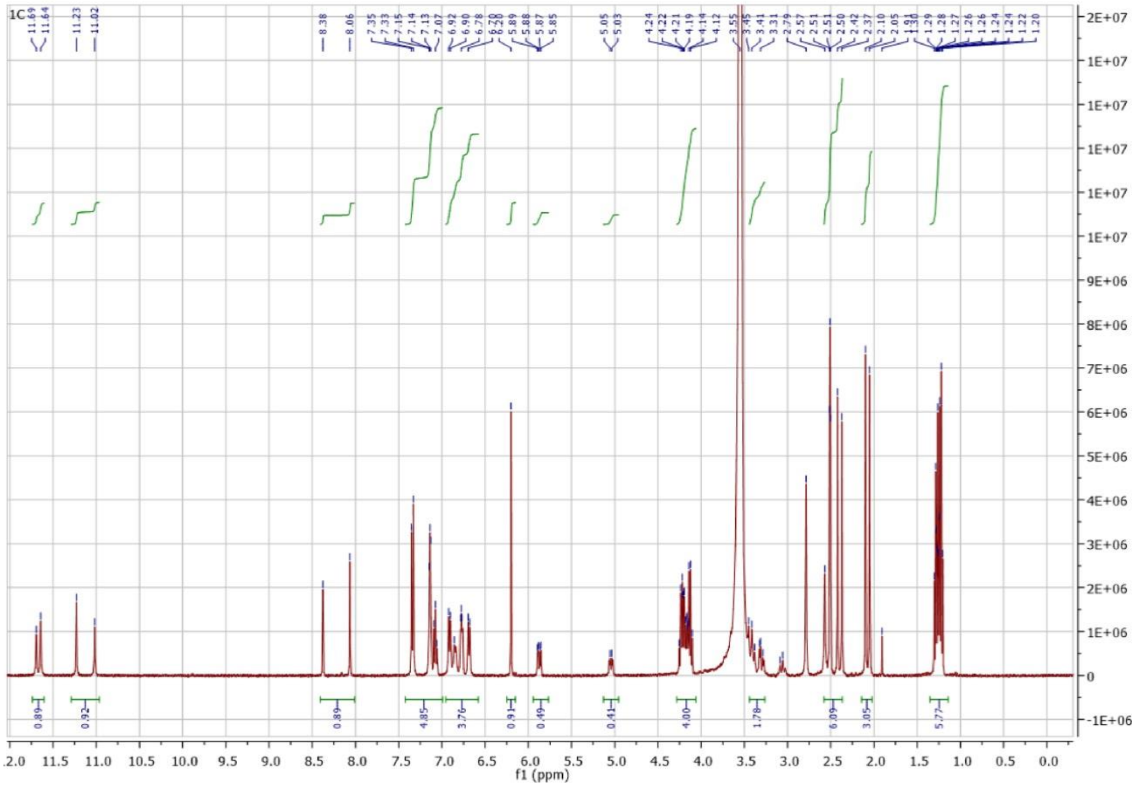

Figure S9. ^{1}H-NMR spectrum of compound 1C

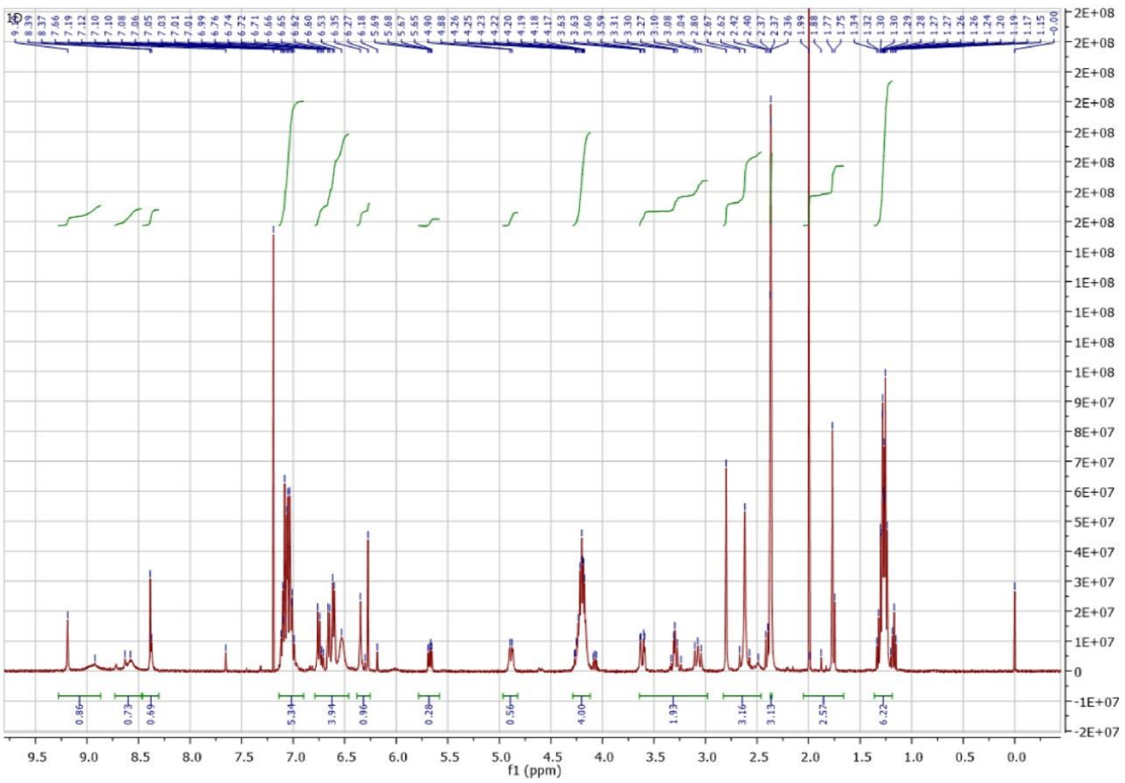

Figure S10. ^{1}H-NMR spectrum of compound 1D

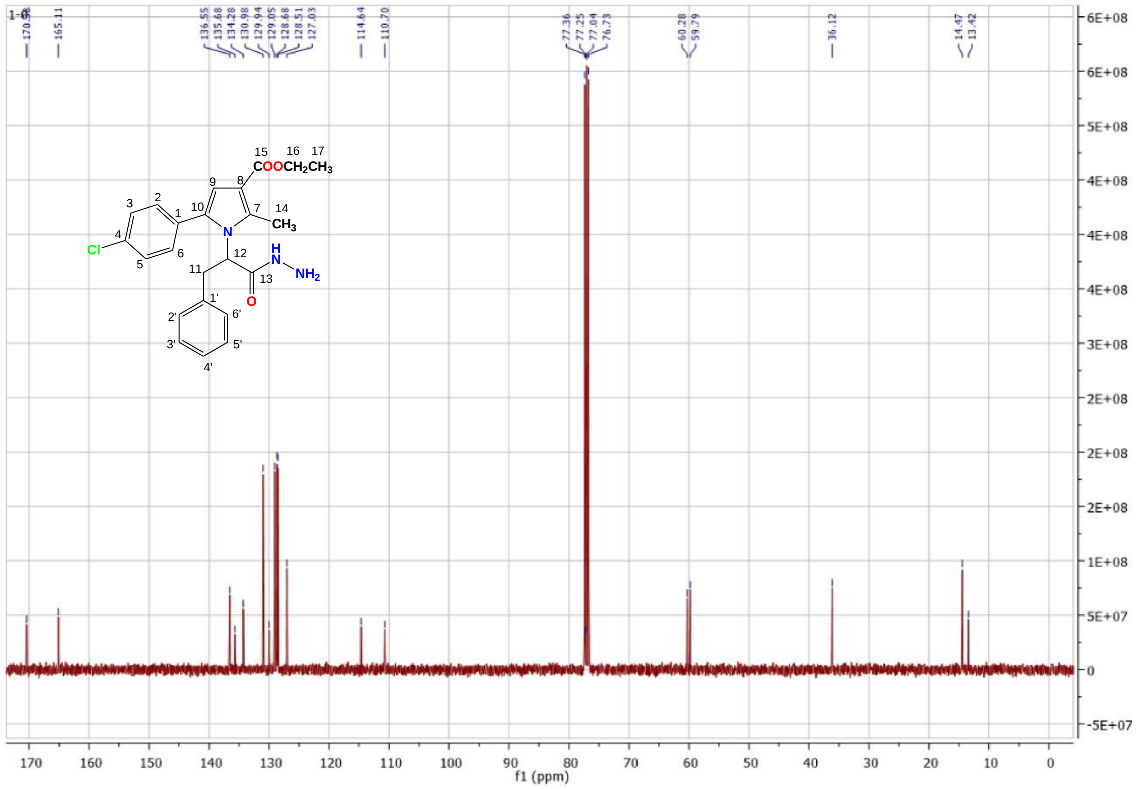

Figure S11. ^{13}**C-NMR spectrum of compound 1**

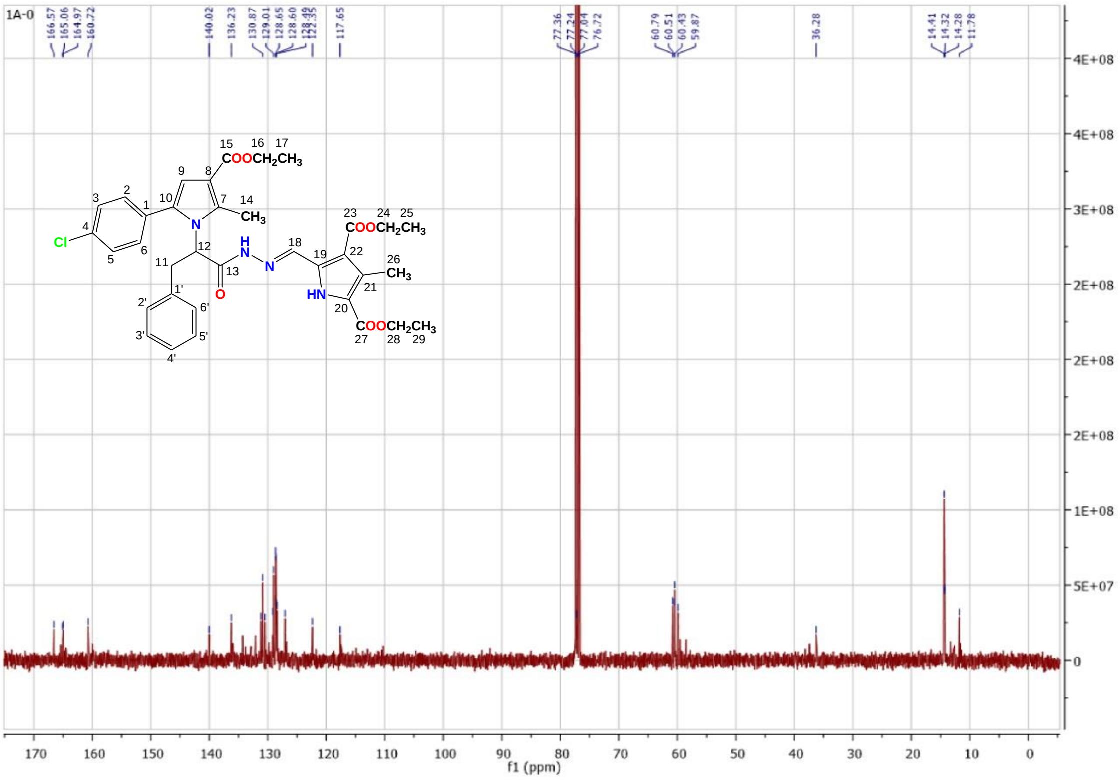

Figure S12. ^{13}**C-NMR spectrum of compound 1A**

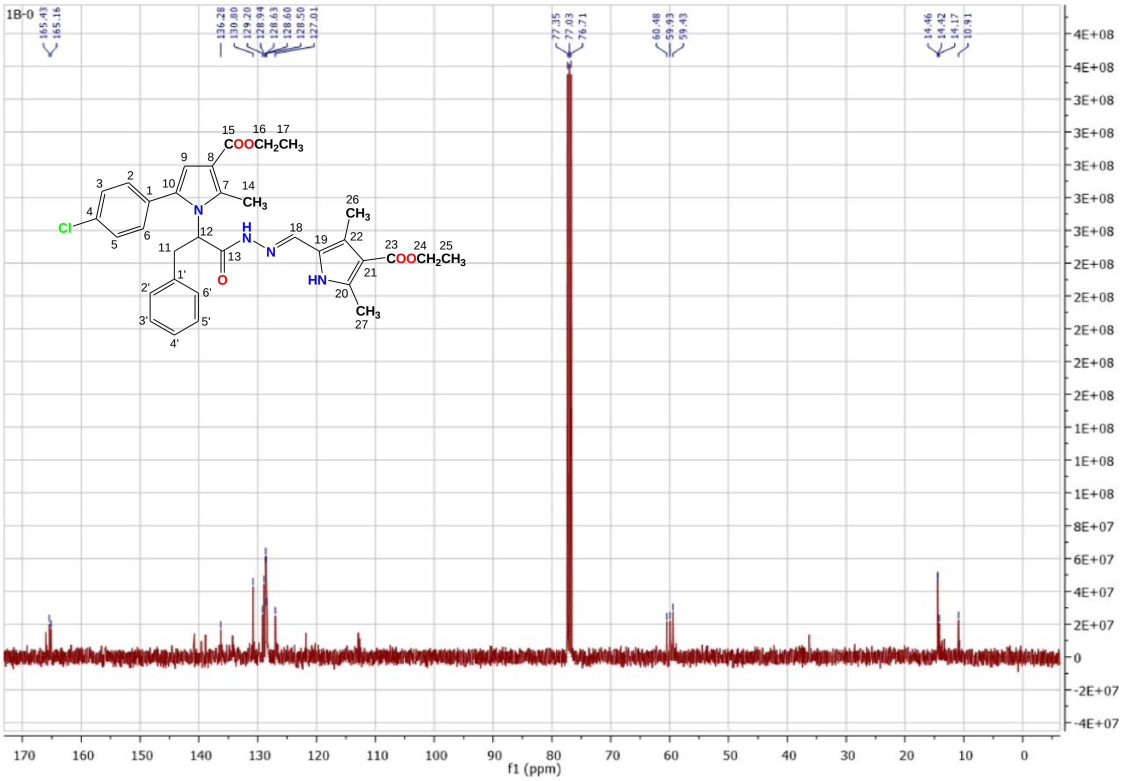

Figure S13. ^{13}**C-NMR spectrum of compound 1B**

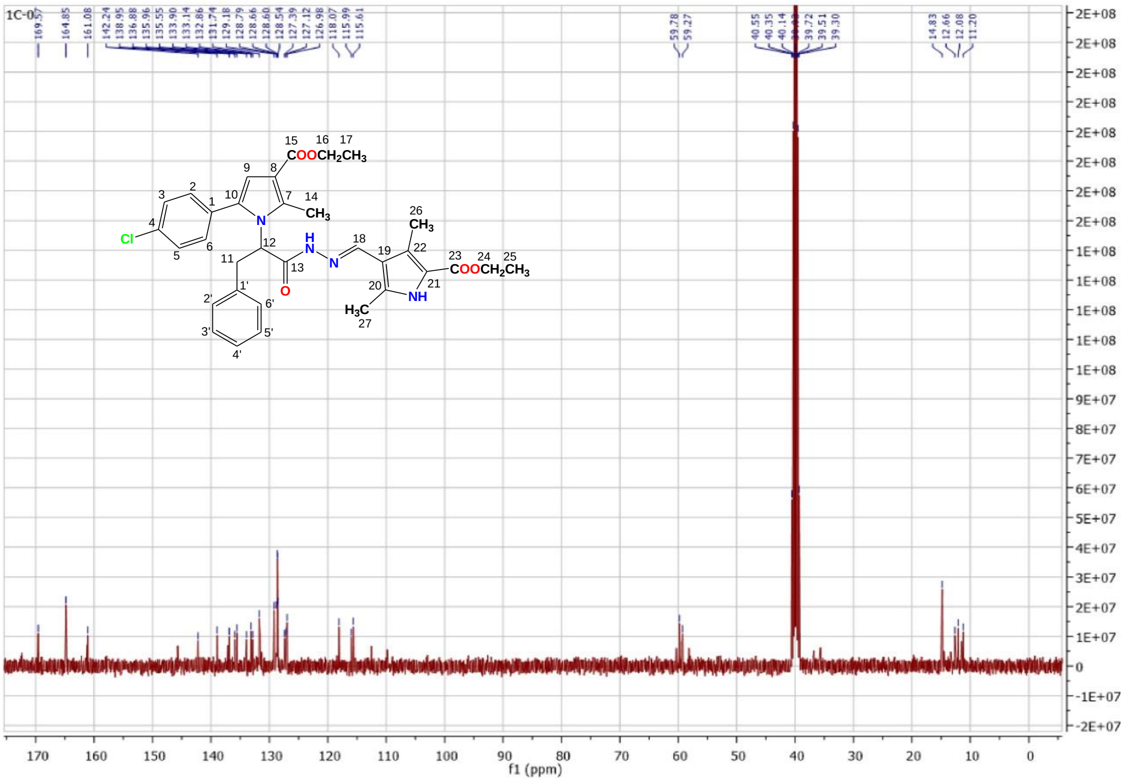

Figure S14. ^{13}**C-NMR spectrum of compound 1C**

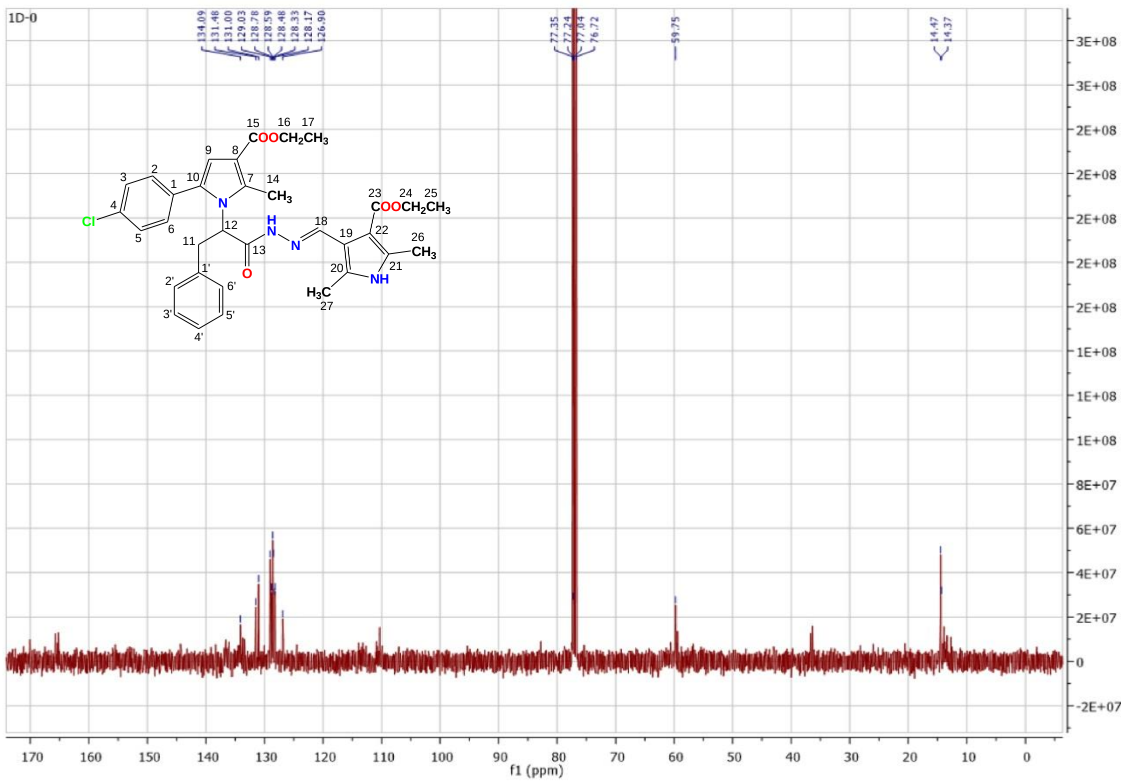

Figure S15. ^{13}C-NMR spectrum of compound 1D

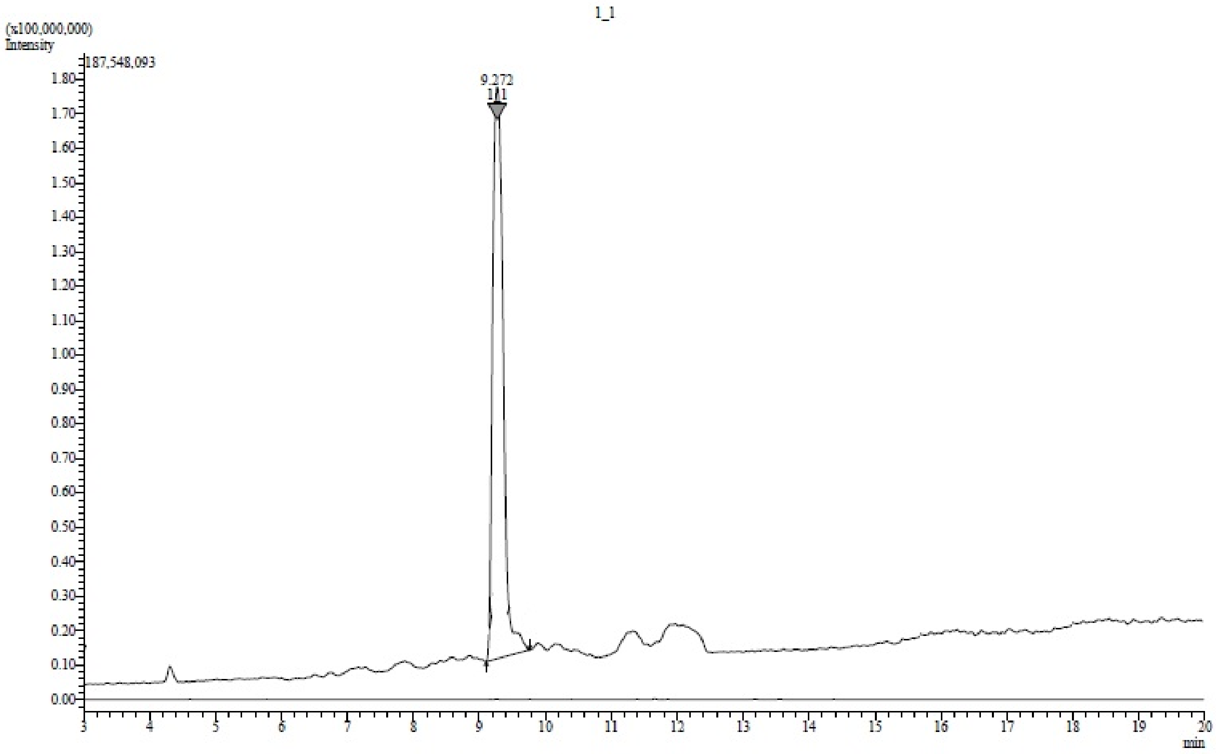

Figure S16. HPLC of compound 1

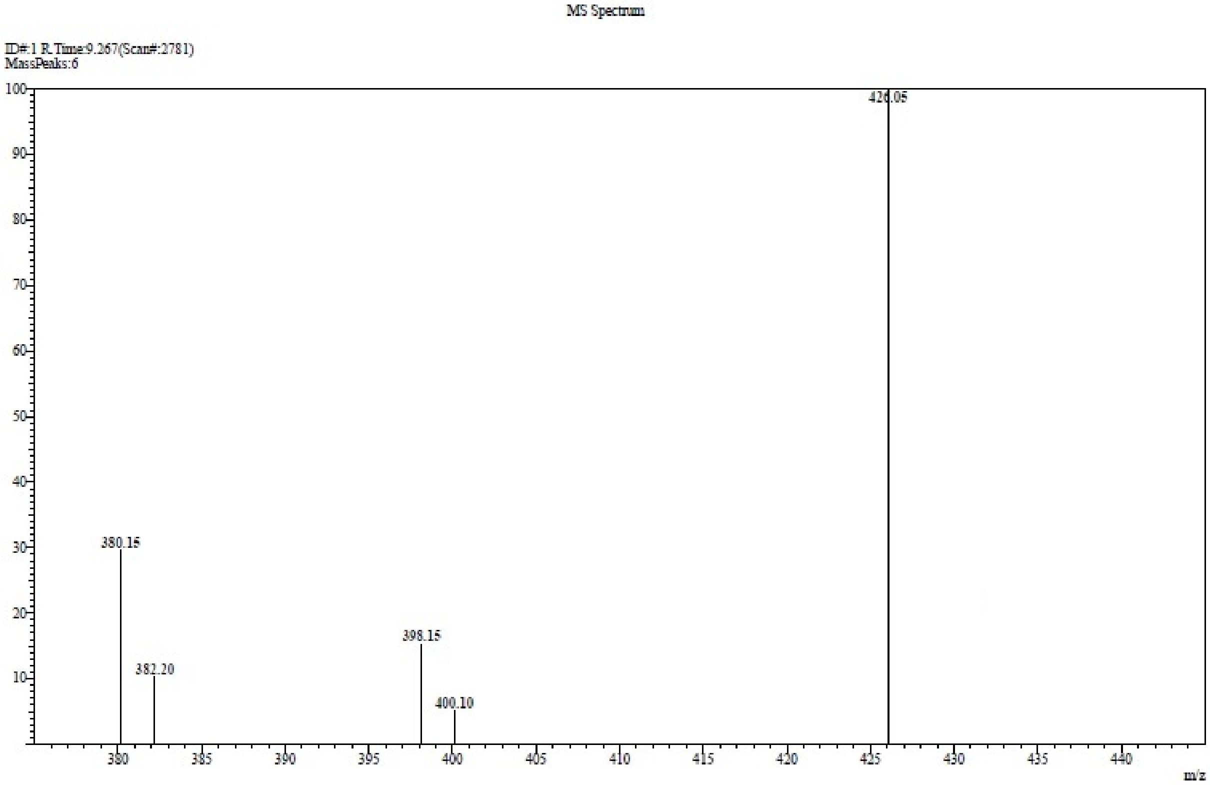

Figure S17. Mass spectra of compound 1

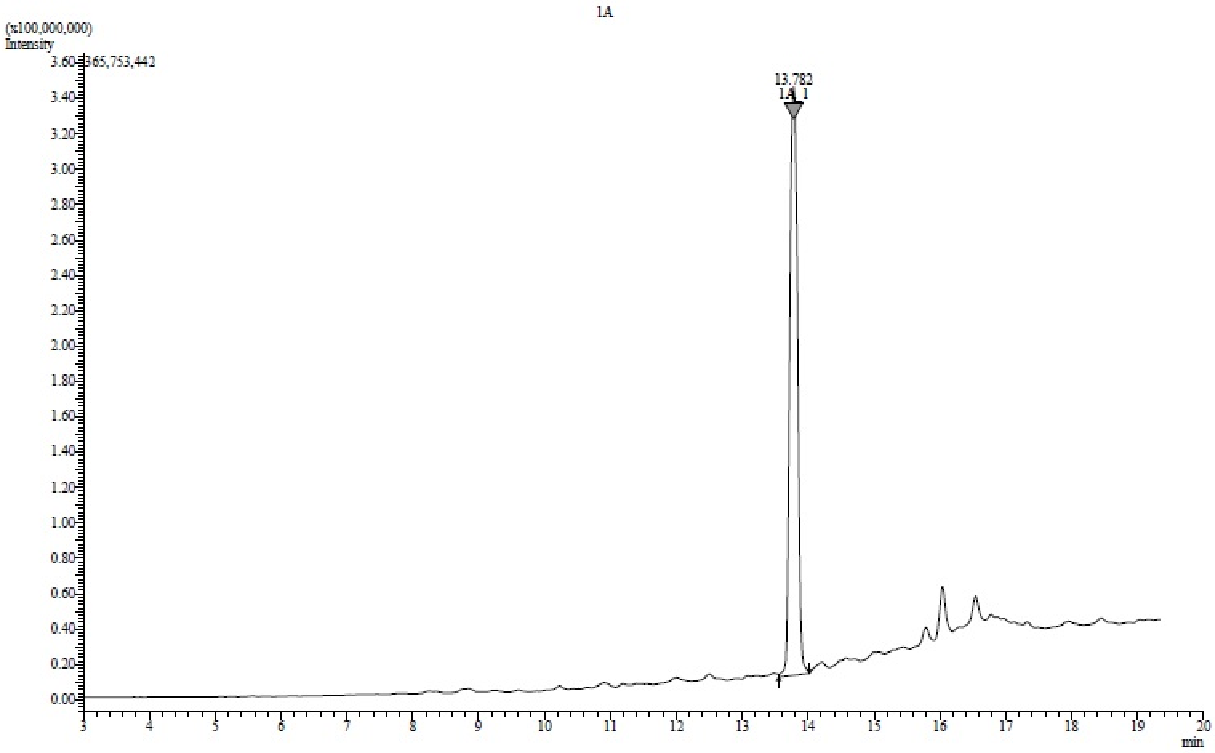

Figure S18. HPLC of compound 1A

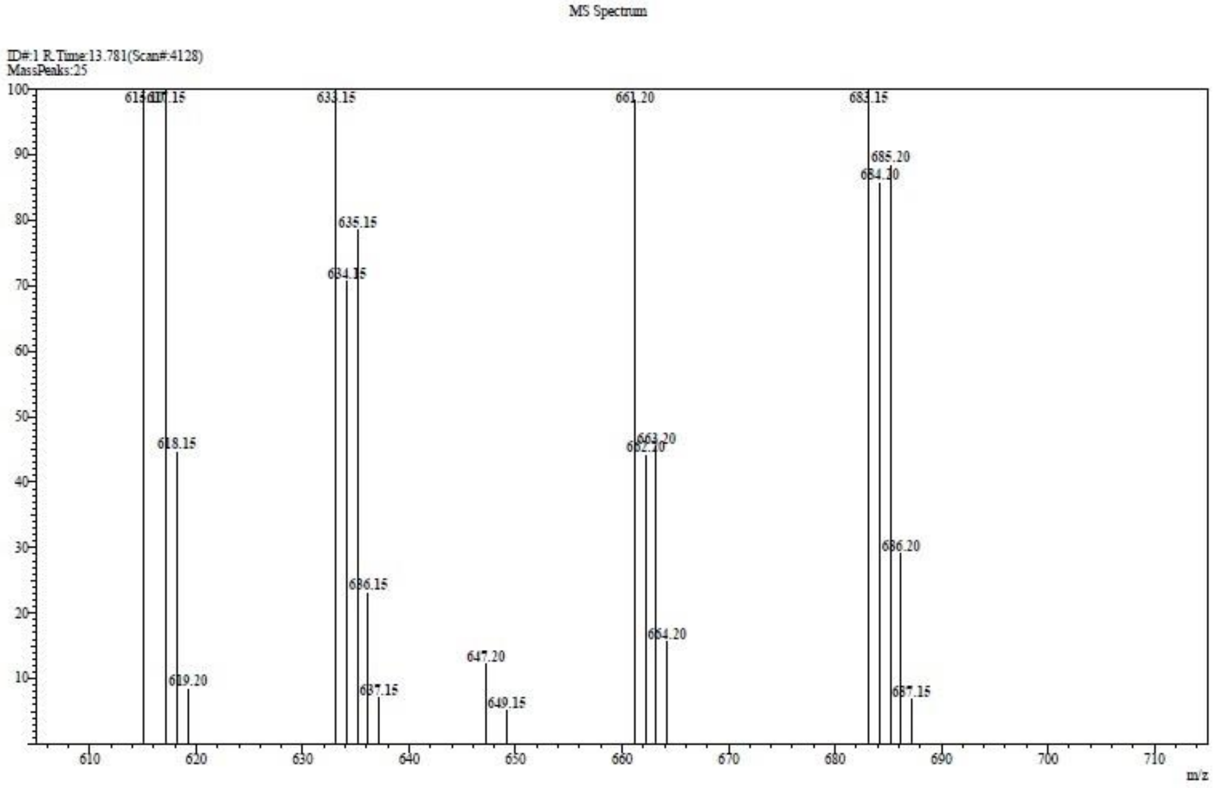

Figure S19. Mass spectra of compound 1A

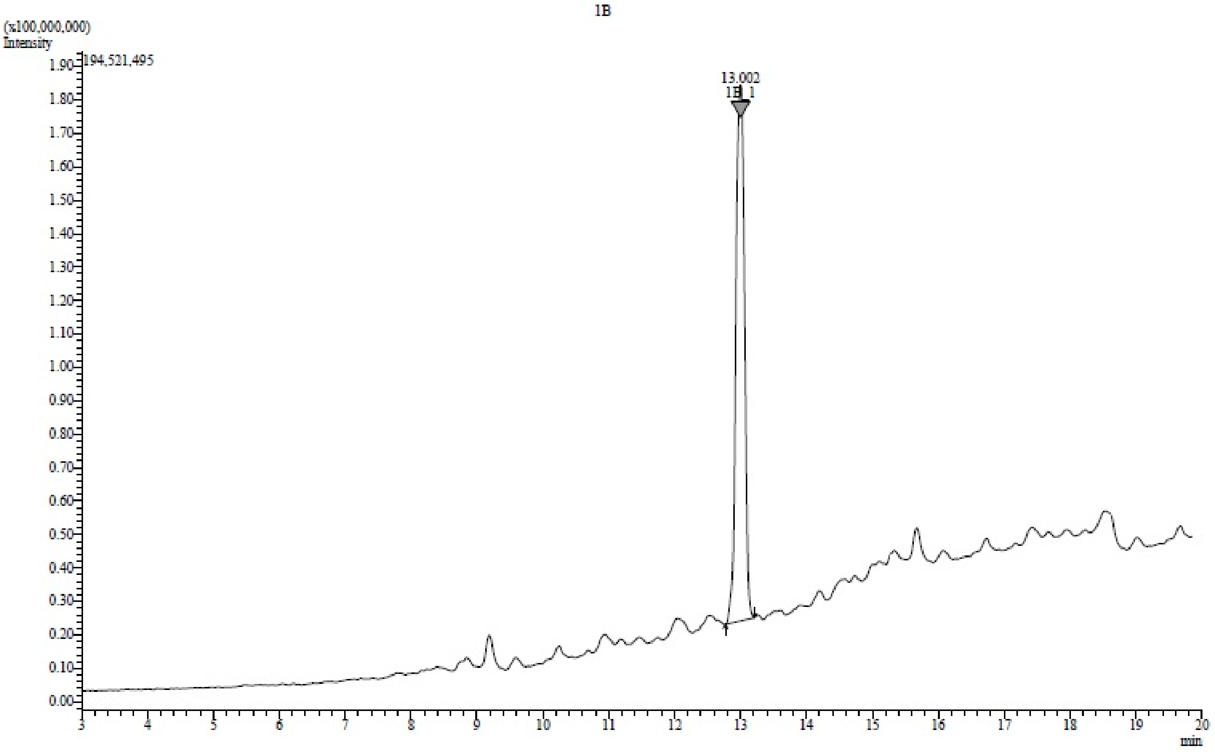

Figure S20. HPLC of compound 1B

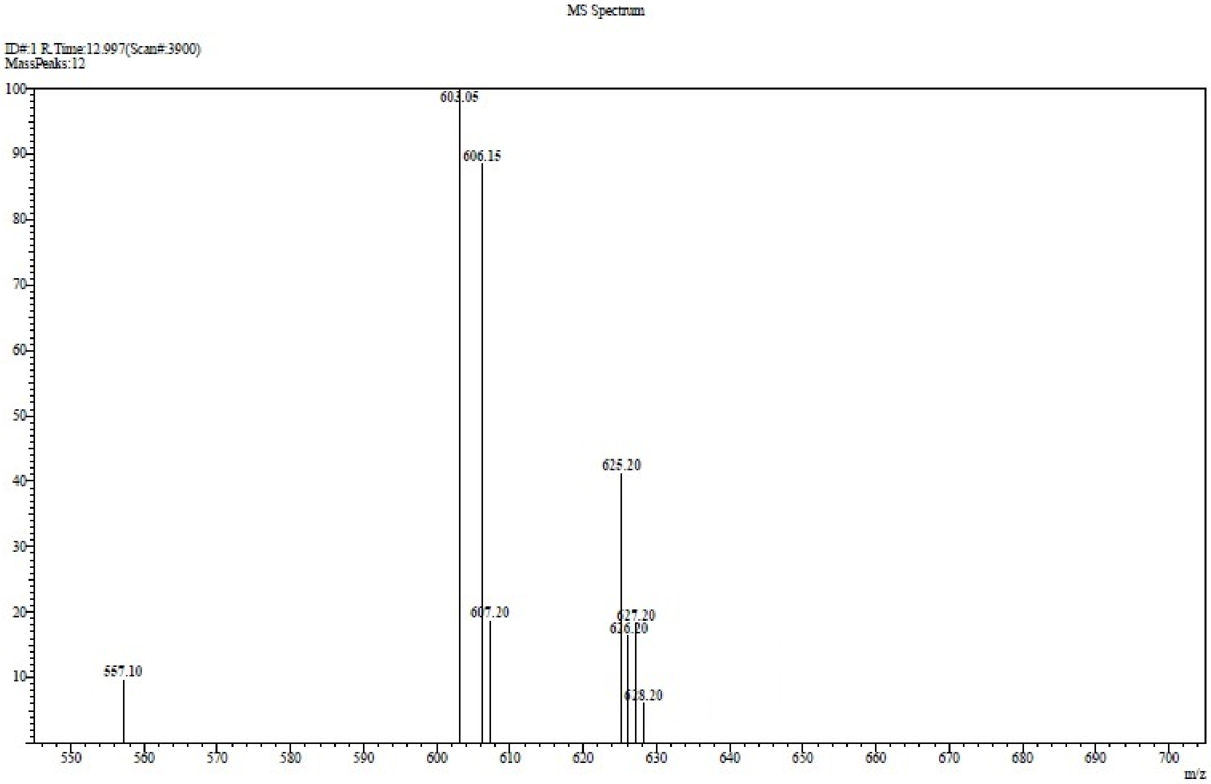

Figure S21. Mass spectra of compound 1B

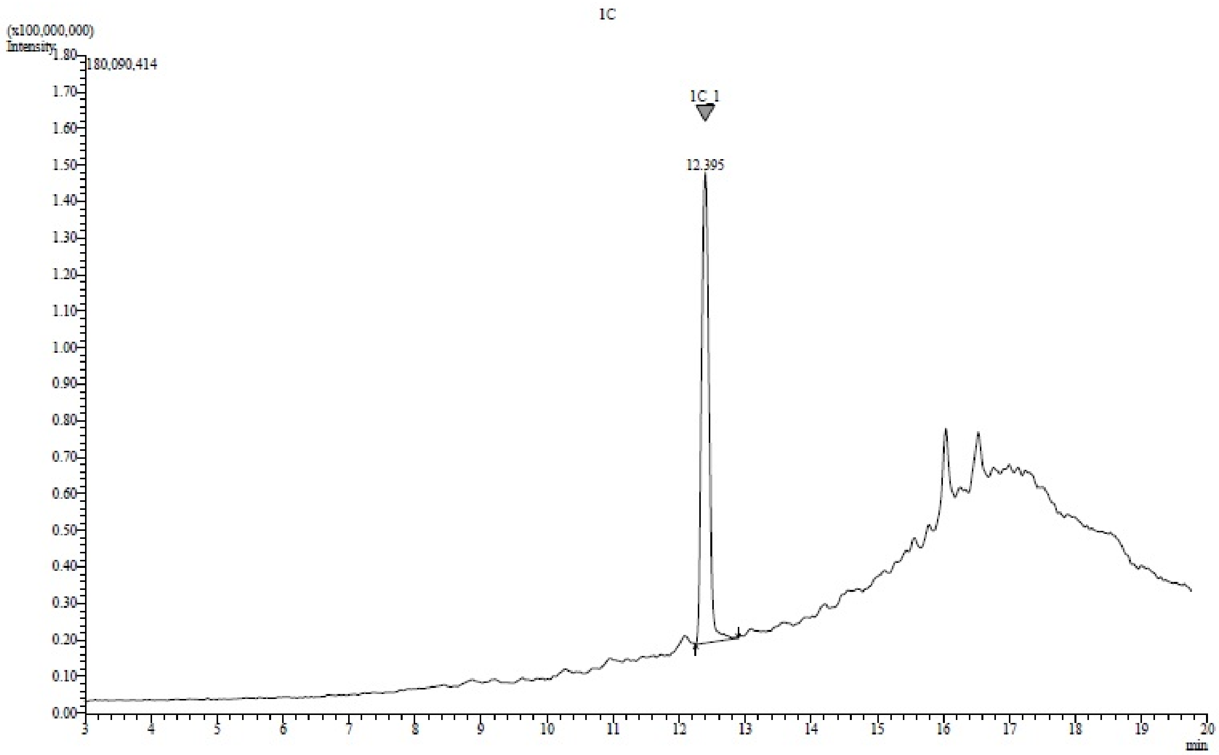

Figure S22. HPLC of compound 1C

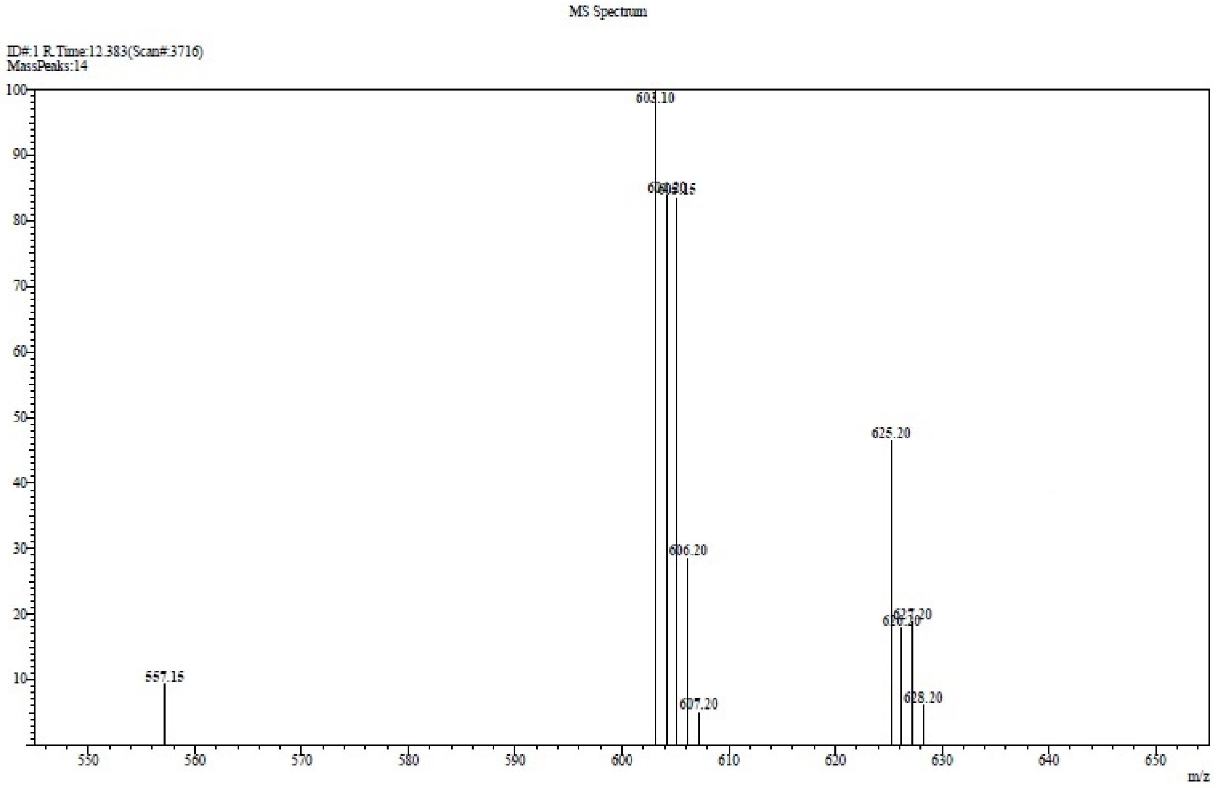

Figure S23. Mass spectra of compound 1C

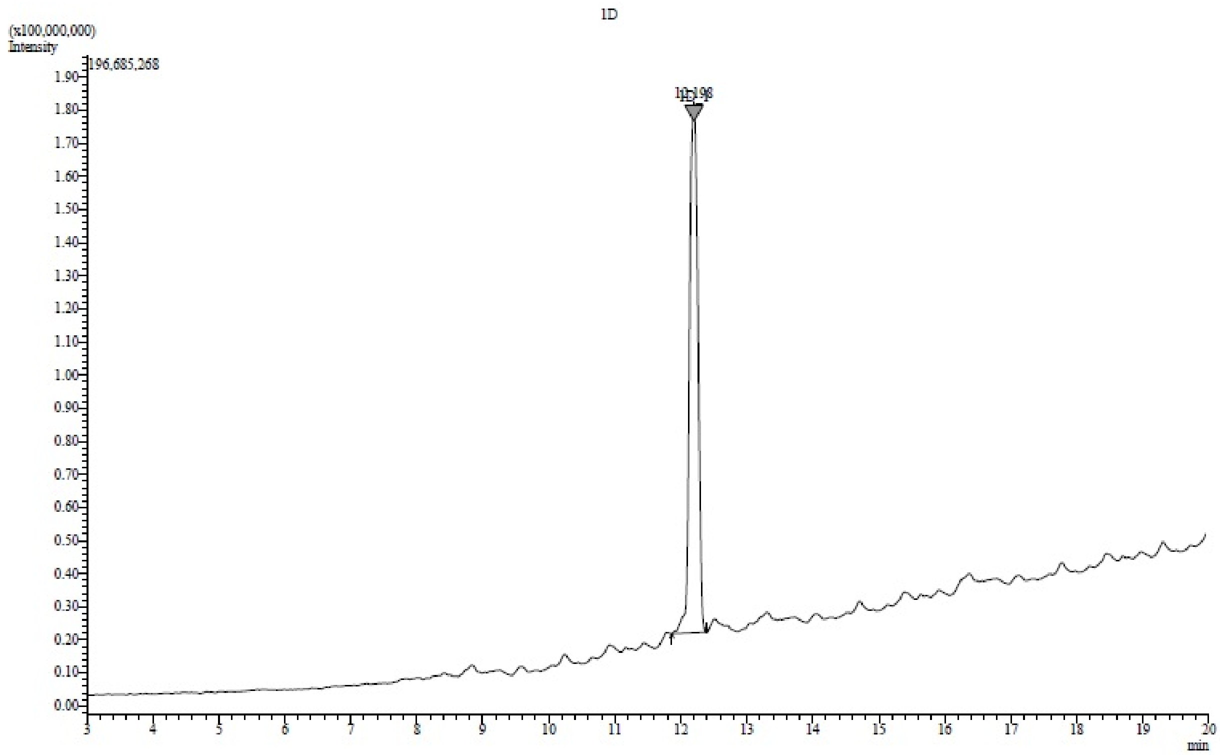

Figure S24. HPLC of compound 1D

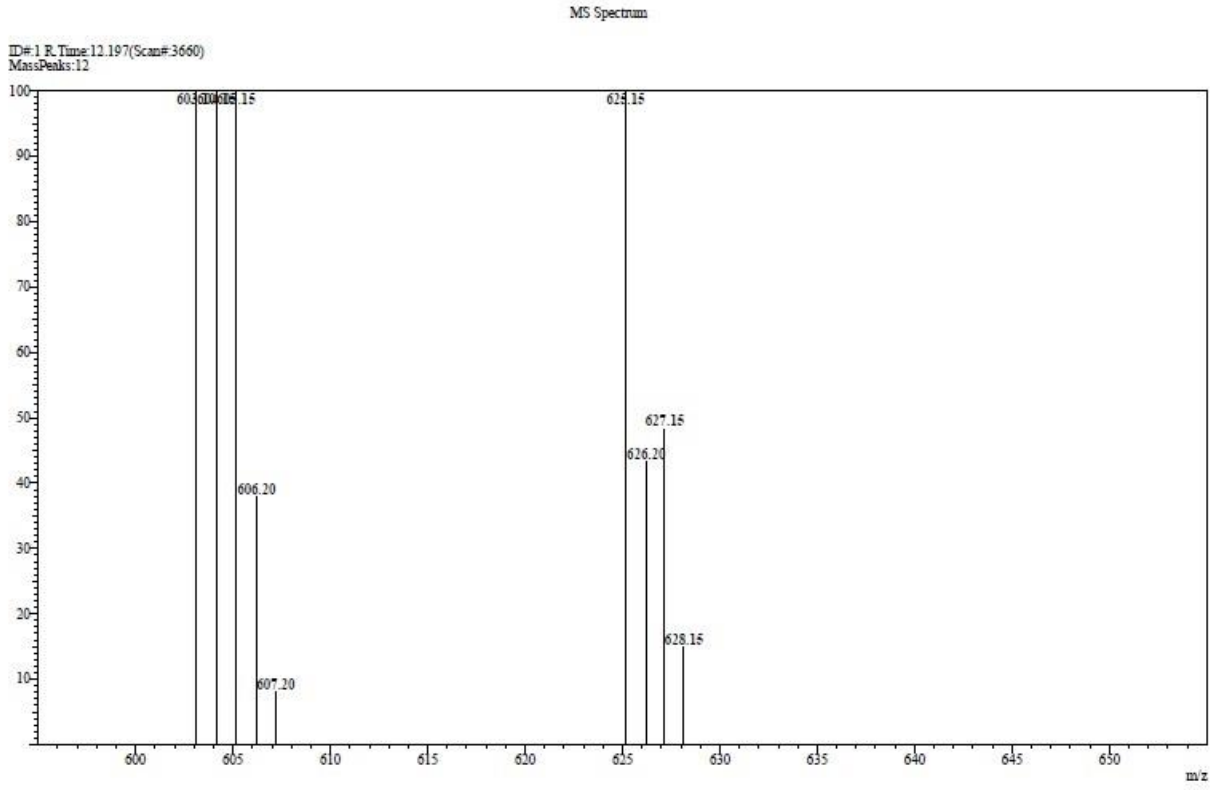

Figure S25. Mass spectra of compound 1D

	Live	Necrosis	Early apoptosis	Late apoptosis
**Control**	99.2	0.19	0.50	0.15
**1**	48.3	0.19	42.7	8.74
**1A**	58.2	0.50	36.5	4.78
**1B**	44.6	0.46	48.9	6.1
**1C**	53.4	0.43	39	7.13
**1D**	60.2	0.78	32.5	6.51
**Cisplatin**	60.7	0.34	36.4	2.59

Table S1. Percentage of cell populations displaying viable and apoptotic trends after treatment with new pyrrole hydrazones conducted via flow cytometry.

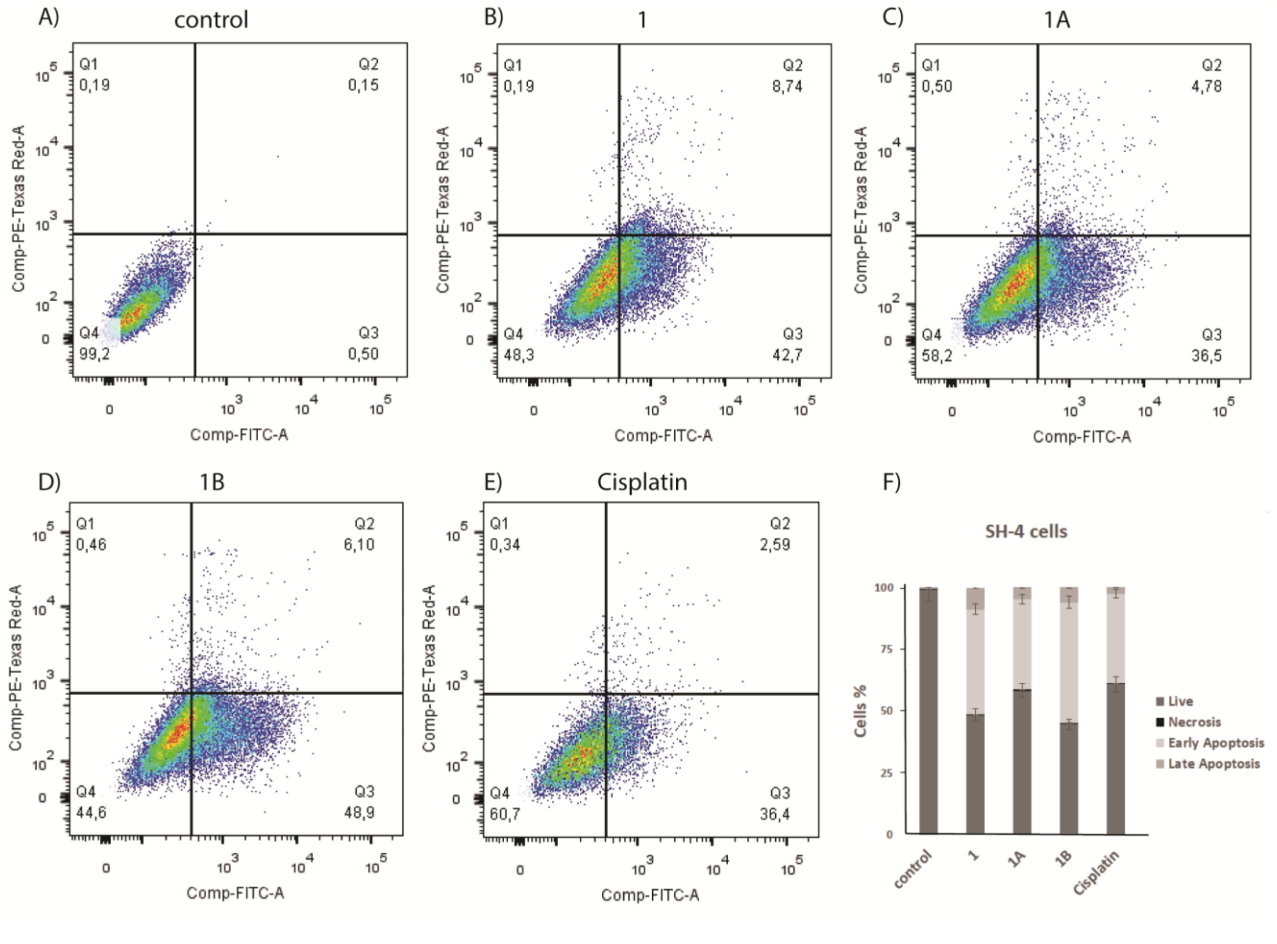

Figure S26. Effect of new pyrrole hydrazones on human cancer cell line SH4 following 48h treatment. A-D) SH-4 cells treated with *6μM* parental compound **1** and its derivatives – **1A** (260 μM) and **1B** (260 μM) were subjected to both Annexin V-FITC and propidium iodide prior to analysis using flow cytometer. E) Cisplatin (20 μM) was used as a positive control. Dot plots representing control and treated cells. F) Scatter plot represents the percentages of necrosis (upper left), late apoptosis (upper right), viable cells (lower left), and early apoptosis (lower right) populations.

	G0/G1 phase	S phase	G2 phase
**Control**	63.6	21.6	12.1
**1**	57	28.5	13.4
**1A**	59.8	24.5	14.8
**1B**	50.2	34	13.6
**1C**	35	38.5	24.5
**1D**	28.3	46.4	23.2
**Cisplatin**	17.8	61.3	17.7

Table S2: The percentage of cells in the G1, S and G2 phases of the cell cycle after treatment with new pyrrole hydrazones conducted via Flow Cytometry Assay.

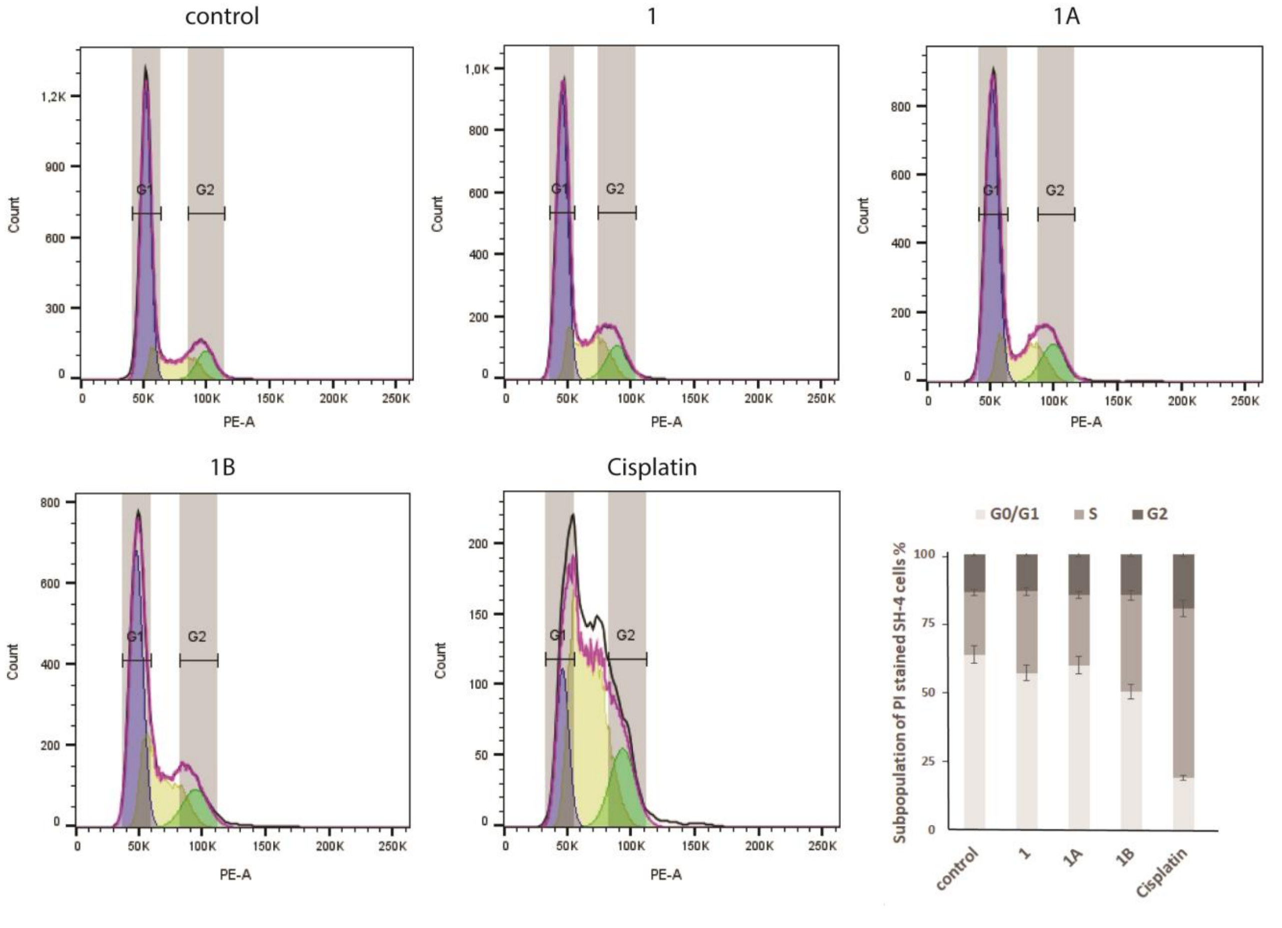

Figure S27. Effect of new pyrrole hydrazones on cell cycle distribution on human cancer cell line SH-4 following 48h treatment. A-D) SH-4 cells untreated (control) or treated with *6μM* parental compound **1** and its derivatives **– 1A (**260μM) and **1B** (260 μM). After treatment, cells were stained with PI and DNA content analyzed by flow cytometry. A representative histogram is shown for each incubation condition. F) Scatter plot represents the percentages of G1/G0, S and G2 populations.
